# Relative contributions of taxonomic and functional diversity to the assembly of plant communities hosting endemic *Dianthus* species in a mountain steppe

**DOI:** 10.1038/s41598-024-56099-x

**Published:** 2024-03-05

**Authors:** Maryam Behroozian, Simon Pierce, Hamid Ejtehadi, Farshid Memariani, Fahime Rafiee, Mohammad Reza Joharchi

**Affiliations:** 1 Herbarium FUMH, Ferdowsi University of Mashhad, Mashhad, Iran; 2 Department of Agricultural and Environmental Sciences (DiSAA), University of Milan, Via G. Celoria 2, 20133 Milan, Italy; 3 Quantitative Plant Ecology and Biodiversity Research Laboratory, Department of Biology, Faculty of Science, Ferdowsi University of Mashhad, Mashhad, Iran; 4 Department of Range and Watershed Management, Faculty of Natural Resources and Environment, Ferdowsi University of Mashhad, Mashhad, Iran

**Keywords:** Biodiversity pattern, Community assembly, Mountain ecosystems, Endemic species, Climatic variables, Soil factors, Ecology, Ecology

## Abstract

Plant community assembly is the outcome of long-term evolutionary events (evident as taxonomic diversity; TD) and immediate adaptive fitness (functional diversity; FD); a balance expected to shift in favour of FD in ‘harsh’ habitats under intense selection pressures. We compared TD and FD responses along climatic and edaphic gradients for communities of two species (*Dianthus pseudocrinitus* and *D. polylepis*) endemic to the montane steppes of the Khorassan-Kopet Dagh floristic province, NE Iran. 75 plots at 15 sites were used to relate TD and FD to environmental gradients. In general, greater TD was associated with variation in soil factors (potassium, lime, organic matter contents), whereas FD was constrained by aridity (drought adaptation). Crucially, even plant communities hosting different subspecies of *D. polylepis* responded differently to aridity: *D. polylepis* subsp. *binaludensis* communities included a variety of broadly stress-tolerant taxa with no clear environmental response, but TD of *D. polylepis* subsp. *polylepis* communities was directly related to precipitation, with consistently low FD reflecting a few highly specialized stress-tolerators. Integrating taxonomic and functional diversity metrics is essential to understand the communities hosting even extremely closely related taxa, which respond idiosyncratically to climate and soil gradients.

## Introduction

The assembly of plant communities represents the outcome of long-term evolutionary histories and the daily functioning and survival of the individuals that comprise the local populations of each species^1^. Evolutionary effects on community assembly can be quantified as local ‘taxonomic diversity’ (TD). Variation in species functioning can be measured in terms of functional traits (i.e. phenotypic characters directly affecting fitness), expressed as ‘functional diversity’ (FD). Functional traits also underpin plant ecological adaptations and determine viable sets of characters, or ‘ecological strategies’^1^. Comparing TD and FD thus provides a means of considering the roles of both ecology and evolution during plant community assembly^1^. Rarely are TD and FD measured simultaneously and their relative importance as determinants of local biodiversity compared, although such studies have recently started to illuminate a range of ecosystems^2–7^. A key ‘natural experimental’ system for comparing TD and FD involves relating these to environmental gradients of abiotic factors (particularly temperature, precipitation, and soil factors^8–10^). However, studies comparing TD and FD environmental responses for plant communities tend to focus on woody vegetation^6,11^, while herbaceous mountain species and their communities are currently represented by the example of *Ionopsidium savianum* (Caruel) Ball ex Caruel (Brassicaceae) in Italy^12^, which is not an endemic or endangered species. Focus is required on the determinants of biodiversity for plant communities hosting endemic and endangered species, particularly for mountain habitats in which driving environmental gradients may be extreme.

A number of environmental factors are known to impact biodiversity and threaten habitats and species^13–15^, and it is evident that climate, soil type, and topography are the most important general factors determining diversity patterns at regional or local scales^16–22^, ultimately determining variation in vegetation composition and the biological diversity and richness of plant communities in mountain regions^23–26^. Thus declining biodiversity in mountain areas is often associated with changing values of climate and soil parameters along gradients^19,27^ and assessments of biodiversity responses are critical to understanding the impacts of environmental changes. Exploring the spatial variation of biodiversity and its relationships with environmental factors is also of general relevance in ecology^14,28,29^ and from a conservation point of view could help to address potential threats to biodiversity, especially for the habitats of endemic and endangered species in mountain ecosystems^21,30,31^.

**Figure 1:**
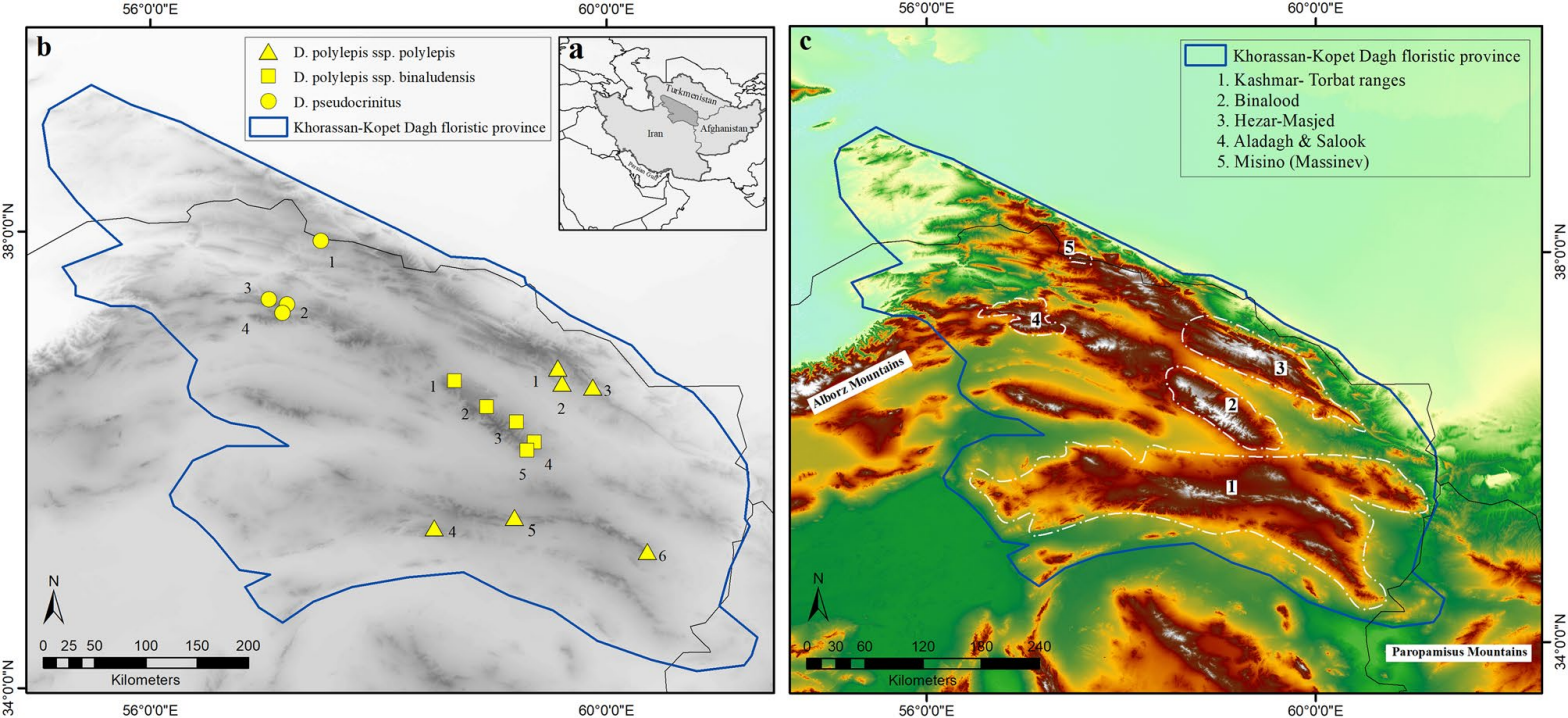
The maps of the study area and sampling sites; (**a**) geographic position of Khorassan-Kopet Dagh foristic province (KK) in northeastern Iran and southern Turkmenistan. (**b**) sampling sites: triangles (*Dianthus polylepis* subsp. *polylepis* sites): S1. Balghur, S2. Kardeh Dam, S3. Khowr, S4. Kuhsorkh, S5. Khomari Pass, S6. Bezd. squares (*D. polylepis* subsp. *binaludensis* sites): S1. Baharkish, S2. Dahane Jaji, S3. Zoshk, S4. Moghan, S5. Dizbad. circles (*Dianthus* *pseudocrinitus* sites): S1. Misino, S2. Biu Pass, S3. Rein, S4. Rakhtian. (**c**) locations of the mountain systems in KK where samplings were carried out. Prepared using ArcGIS 10.3 software (http://www.esri.com).

Here, we selected two *Dianthus* species (*D. pseudocrinitus* Behrooz. & Joharchi and *D. polylepis* Bien. ex Boiss.) endemic to the montane steppes of the Khorassan-Kopet Dagh floristic province (KK) of northeastern Iran (Fig. 1; locations detailed in Table S1) to assess the effect of environmental factors on biodiversity indices in the communities of these species. These taxa occur in mountain habitats with severe environmental conditions and along with their communities they can reasonably be expected to be sensitive to environmental changes. The communities of these taxa can thus be important targets for conservation activities due to being affected and threatened by human activities, climate change, and topographic barriers^15,32,33^. *Dianthus pseudocrinitus* is a narrow endemic plant species with particular habitat requirements, restricted to the Aladagh, Salook, and Massinev mountains in the KK (Fig. 1c). The species is usually found in patches in montane steppes where environmental stressors are prevalent and the majority of species are adapted to stress, and this species can alter its ecological strategy from stress-tolerant to ruderal depending on the local extent of disturbance^15^. Indeed, these habitats are threatened by anthropogenic disturbances such as overgrazing, road building, agriculture and subsequent habitat fragmentation, which are inducing changes particularly in soil characteristics^15,34^. Another endemic species, *Dianthus polylepis*, has a broader geographic distribution, throughout the KK floristic province, and includes two formally recognized subspecies: *D. polylepis* subsp. *polylepis* and *D. polylepis* subsp. *binaludensis* (Rech.f.) Vaezi and Behrooz. with mostly disjunct geographic ranges. *D. polylepis* subsp. *binaludensis* is restricted to the Binalood Mountains, characterized by successions of sedimentary, metamorphic, and igneous rock^35^, whereas *D. polylepis* subsp. *polylepis* is distributed broadly in the calcareous Khorassan-Kopet Dagh mountains^36^. The subspecies of *D. polylepis* often occur in stressful habitats of rocky slopes, which have become affected by human activities such as road building. Their habitats are influenced by high temperatures and low precipitation, especially within habitats of *D. polylepis* subsp. *polylepis* in the southern mountain ranges of the KK. These ecological contrasts between habitats of two species allow us to compare biodiversity patterns and their relationships with the different environmental influences such as climate, topography, and soil factors.

Therefore, we studied the taxonomic and functional alpha diversity of the communities hosting the two endemic *Dianthus* species (including subspecies) along various environmental gradients in mountain steppes, demonstrating the relative importance of climate and soil factors on structuring different aspects of biodiversity in these communities. Specifically, we addressed the following hypotheses: (i) differential variation in taxonomic and functional diversities occurs along climatic and soil factor gradients, with characteristic TD/FD patterns for the communities hosting each *Dianthus* species, (ii) adaptive specialization (low FD) is particularly evident in the harshest, arid habitats, converging on stress-tolerance. Our findings may allow conservation biologists to target species conservation more effectively by providing a mechanistic assessment of the context in which species survive. Support for these hypotheses would suggest that the conservation of endemic species cannot assume that the plant communities hosting closely related species will respond in similar ways to environmental gradients.

## Materials and methods

### Study area

This study was conducted within the Khorassan-Kopet Dagh floristic province (KK) in northeastern Iran (34° 20′ to 39° 13′ N and 55° 05′ E to 61° 20′ E) (Fig. 1a,b). The Kopet Dagh range includes the high peaks of Allaho-Akbar and Hezar-Masjed mountains. Northern ranges of Khorassan comprise the mountains of Ghorkhod, Aladagh, Salook, Shah-Jahan, and Binalood, whereas the Sabzevar and Kashmar-Torbat ranges are oriented mainly east–west at the southern border of the KK floristic region (Fig. 1c). Most of the KK region is characterized by a Mediterranean or Irano-Turanian xeric-continental bioclimate, except for high montane areas in the central KK, where a Mediterranean or Irano-Turanian pluvi-seasonal continental bioclimate is evident, with shorter summer drought and higher annual precipitation^37,38^. Mean annual temperature ranges from 12 to 19 °C, depending on elevation^39^; mean annual precipitation is 175–300 mm on the plains and foothills and 300–380 mm in montane regions.

### Site selection

Fieldwork was undertaken in different habitats across the ranges of *D. pseudocrinitus* and *D. polylepis* during three successive years (2016–2018), in the mountain steppes of northeastern Iran. A total of 15 sites were selected based on geographic distances and contrasting ecological conditions across different ranges of Binalood mountains for *D. polylepis* subsp. *binaludensis*, Hezar-Masjed and Kashmar-Torbat ranges for *D. polylepis* subsp. *polylepis*, and Misino to Aladagh-Salook ranges for *D. pseudocrinitus* (Table S1; Fig. 1b,c). At each site, five 5×5 m plots were established, and GPS coordinates were recorded for longitude, latitude, and elevation. Canopy cover, species abundance, as well as soil and topographic information were recorded in each quadrat. In total, 75 plots were sampled in the study area, covering a significant portion of the geographic ranges of these two endemic *Dianthus* species. The vascular plant species occurring within all quadrats were collected to identify and measure functional traits in each plot (Table S2).

### Plant functional information

We determined three quantitative traits to assess functional diversity, including leaf area (LA), leaf dry-matter content (LDMC), and specific leaf area (SLA) (traits highly representative of plant resource economics and size variation^40–43^) for all species of each quadrat with a cover ≥ 5%, following the leaf area and weight measurement methodologies^44^. The material was collected from April to early July 2016–2018, when leaves were fully expanded and mature. We selected only the most prevalent species (≥ 5% cover), because of their greater influence on ecosystem processes^45–47^ and the impracticality of performing a fully replicated analysis of functional traits that included all subordinate and transitory species. Ten individuals of each species were collected, packed in moist paper bags, sealed in plastic bags, and stored in a thermal box until storage at 4 °C for 12–24 h. Depending on the size of the leaves, 2–10 undamaged, fully expanded young leaves (including the petiole) were measured per individual. We determined the leaf area using a digital scanner and Leaf Area Measurement v1.3 software (Andrew Askew, University of Sheffield, UK). Turgid leaf fresh weight (LFW) was obtained from saturated leaves, and leaf dry weight was determined after drying for 72 h in an oven at 70 °C.

Plant material was sampled in accordance with the IUCN Position Statement^48^: (1) research was led by professional scientists from the country where the species occurs, (2) plant material, consisting of leaves and not entire plants, was collected non-lethally, (3) plant material was collected in accordance with national laws governing the conservation of rare species, (4) plant material was collected responsibly, with the ultimate aim of improving knowledge of the species and their habitats to aid conservation (this included collecting the minimum amount of material to address the research objectives, and prior determination that material was not available from museums or other institutional collections). Note that the *Dianthus* species investigated in the present study have not been assessed according to IUCN protocols and are thus not included in the IUCN Red List (https://www.iucnredlist.org), and do not have an ‘official’ classification of the extent of rarity. Note also that the present study did not require the transport of plant material across international borders, and thus the Convention on International Trade in Endangered Species of Wild Fauna and Flora (CITES; https://cites.org/eng) was not applicable.

### Environmental factors

Soil type, topography, and climate were recorded for each quadrat. The surface soil samples (i.e., upper 15–25 cm of topsoil) were collected for each quadrat, placed in a polyethylene bag, labeled, and transported to the laboratory. Soil texture was measured by three-fraction particle sizes (sand, silt, and clay) using Bykas’ hydrometric method^49^. Acidity (pH), electrical conductivity (EC), cation-exchange capacity (CEC), organic carbon, organic matter, total nitrogen, potassium (K), phosphorus (P), and lime (calcium carbonate; CaO_3_) percentage were also measured for each sample. Total nitrogen (N) was determined by the Kjeldahl method^50^. Organic carbon (OC) was analyzed by the Walkley and Black method^51^. Soil electrical conductivity (EC) and acidity (pH) were determined using pH and EC meters. Total potassium (K) was analyzed by flame atomic absorption spectrophotometry^52^. Absorbable phosphorus was analyzed by the Olsen method^53^. The percentage of total lime was measured by titration with 0.01 N NaOH^54^. Bioclimatic variables were extracted from the WorldClim global climate database, with a 30″ spatial resolution^55^. The latitude and longitude of each quadrat were recorded in the software ArcGIS 10.3.1., and corresponding values of the bioclimatic variables were extracted for each quadrat site.

### Analysis of data and statistical testing

The taxonomic diversity was measured using the first three Hill numbers to estimate species richness (q = 0), the exponential of Shannon’s entropy (q = 1; referring to Shannon diversity), and the inverse of Simpson’s concentration (q = 2; referring to Simpson diversity). The analysis was computed using the R package *hillR*^56^. We used the community-weighted mean (CWM) index and multi-trait functional diversity indices such as FRic (functional richness), FEve (functional evenness), FDiv (functional divergence), FDis (functional dispersion), and RaoQ (Rao’s quadratic entropy) to calculate the functional diversity of the communities of the *Dianthus* taxa. The community-weighted means were calculated for each trait and community sample and were considered as mean trait values for each vegetation plot, weighted by the relative abundances of species with particular trait values^57,58^. We also applied CSR (Competitiveness, Stress-toleration, Ruderality) scores obtained by Behroozian et al.^15^ (using the method and classification tool of Pierce et al.^40^) to assess the impacts of environmental factors on survival (CSR) strategies of species at the sites of the three taxa. Community-weighted mean values for functional traits (LDMC, SLA, LA) and C, S, and R scores as well as multi-trait functional diversity indices were calculated for each site using the function “FD” in the R package *FD*^59,60^.

All statistical analyses were performed using the R statistical environment^61^. We analyzed variation in two aspects of biodiversity (taxonomic and functional diversity) within the sites with communities of both endemic *Dianthus* species. Kruskal–Wallis tests were applied using the R package *ggpubr*^62^ to test the significant differences between biodiversity indices across all study sites. Taxonomic and functional diversity relationships with environmental variables and their interactions were analyzed. To avoid highly correlated environmental variables, we used Pearson correlation^63^, and removed one of each pair of variables with a correlation ≥ 0.8. Furthermore, variance inflation factors (VIF) of variables were used amongst the remaining variables using function *vif()* in the package *car*^64^ to quantify how much a regression coefficient was inflated by the presence of other explanatory variables. Collinear environmental variables with high variance inflation factors (> 10) were eliminated from further analyses. Eight variables (clay, pH, EC, K, aspect, Annual Mean Temperature, Annual Precipitation, Precipitation of Driest Quarter), ten variables (clay, pH, EC, organic matter, lime, aspect, Annual Mean Temperature, Annual Precipitation, Isothermality, Temperature Seasonality) and nine variables (EC, P, organic matter, lime, aspect, Annual Mean Temperature, Annual Precipitation, Precipitation of Wettest Month, Precipitation of Wettest Quarter) were selected for *D. polylepis* subsp. *binaludensis*, *D. polylepis* subsp. *polylepis* and *D. pseudocrinitus*, respectively.

As an initial procedure to help determine the main parameters affecting plant variability and thus create specific regressions between plant and climate factors, we applied a forward selection procedure using the function “forward.sel()” in the package *packfor()* to identify the significant environmental variables in the most parsimonious model based on the adjusted coefficient of multiple determination (R^2^_adj_) calculated using all explanatory variables^65,66^ for taxonomic and functional diversity. Accordingly, annual precipitation (bio12) and potassium (K) were selected as the most important factors influencing the plant biodiversity indices in the communities of *D. polylepis* subsp. *binaludensis*, whereas annual precipitation (bio12), and lime and organic matter (OM) were selected in the communities of *D. polylepis* subsp. *polylepis* and *D. pseudocrinitus*, respectively.

Finally, we developed linear regression models with annual precipitation (bio12), potassium (K), lime, and organic matter (OM) factors as explanatory variables and q_0_, q_1_, q_2_, FRic, RaoQ, CWM_LDMC_, CWM_SLA_, CWM_LA_, CWM_C_, CWM_S_, CWM_R_ as response variables^18^. If the relationship between the independent and dependent variables is in the form of a non-linear function with respect to the parameters, the estimation of the model parameters can be obtained with the help of non-linear regression. Linear regression is a simpler model and uses only one independent variable for prediction, while nonlinear regression can use multiple independent variables and more complex transformations for prediction. Moreover, non-linear trends of biodiversity facets with our explanatory predictors were examined by developing non-linear regression models. Therefore, these models were compared using second-order Akiake information criteria (AIC) and R^2^_adj_ values in the communities of three taxa. Finally, we plotted the best models and then obtained R^2^_adj_ values using the package vegan^67^.

Furthermore, we carried out variation partitioning (VP) based on partial linear regression using the “varpart” function^67^ to quantify the relative importance of precipitation and soil factors and their interaction on biodiversity indices at the sites of both species. The total percentage of variation explained was divided into unique and shaded contributions for two sets of predictors: (i) precipitation (white fraction), (ii) soil (green fraction; potassium at the sites of *D. polylepis* subsp. *binaludensis*, and lime at the sites of *D. polylepis* subsp. *polylepis* and organic matter at the sites of *D. pseudocrinitus*), and (iii) shared contributions of both factors (shared area between white and green fractions). Analyses were performed in R ver. 4.2.2, and figures were created by the *ggplot2* package^62^.

## Results

### Plant taxonomic and functional diversity among the *Dianthus* communities

**Figure 2:**
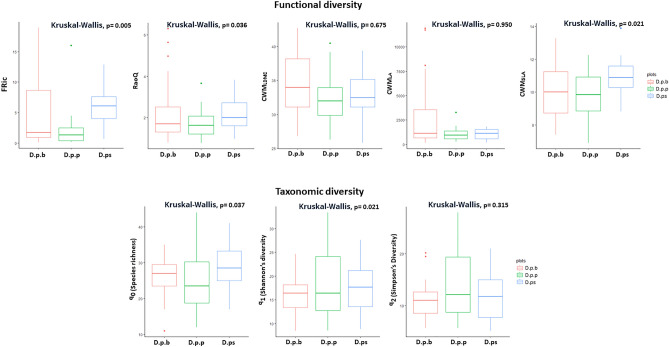
Hill’s diversity (q_0_—Species richness, q_1_—Shannon’s diversity, q_3_—Simpson diversity), Functional (RaoQ, FRic, CWM_SLA_, CWM_LA_, CWM_LDMC_) diversity at plot scale among the communities of *Dianthus* taxa (D.p.b: *D. polylepis* subsp. *binaludensis*; D.p.p: *Dianthus polylepis* subsp. *polylepis*; D.ps: *Dianthus pseudocrinitus*). The lines represent the first and fourth quartiles, the box represents the second and third quartiles and the line within the box represents the median. The points outside of the boxplot represent outliers. Kruskal–Wallis tests followed by Dunn’s post hoc tests with Bonferroni correlation indicate significant differences (as *p < 0.05, **p < 0.01, ***p < 0.001) across the all studied communities.

Among taxonomic diversity indices, Species Richness (q_0_) and Shannon’s diversity (q_1_) indicated significant variation between communities of the three taxa (Fig. 2; q_0_ = Kruskal–Wallis H p = 0.037, q_1_ = Kruskal–Wallis H p = 0.021), such that higher taxonomic diversity was observed in plots of the communities of *D. pseudocrinitus* than plots of communities of *D. polylepis* subsp. *binaludensis*. However, these differences were stronger in plots of *D. polylepis* subsp. *polylepis* with lower species richness (q_0_) than the other communities (Fig. 2). Boxplots of functional diversity also revealed significant differences in FRic (Kruskal–Wallis H p < 0.005), RaoQ (Kruskal–Wallis H p = 0.036), and CWM_SLA_ (Kruskal–Wallis H p = 0.021) between the communities. Thus, values of these indices were highest for the communities hosting *D. pseudocrinitus*. In contrast, the lowest values of functional indices were evident for the communities of *D. polylepis* subsp. *polylepis*, with little difference for the communities of *D. polylepis* subsp. *binaludensis*. In total, the highest values of functional diversity were evident for CWM_LDMC_ and CWM_S_ (i.e., the community weighted mean extent of stress-tolerance) indices when compared with other functional indices for all communities of the *Dianthus* taxa.

### Effects on precipitation and soil factors on TD and FD among *Dianthus* communities

**Figure 3:**
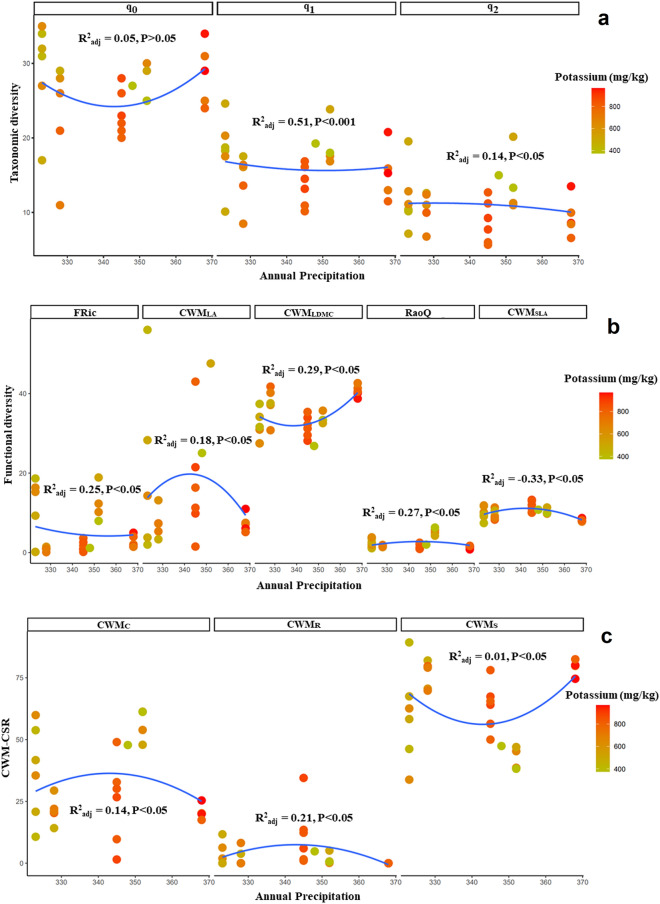
Alpha diversity of communities hosting *D. polylepis* subsp. *binaludensis*: (**a**). taxonomic diversity (species richness, q_0_; Shannon’s diversity, q_1_, Simpson’s diversity, q_2_), (**b**). functional diversity (FRic, RaoQ, CWM_LDMC_, CWM_SLA_, CWM_LA_), and (**c**). plant ecological strategy diversity (C, S, R strategies; CWM_C_, CWM_S_, CWM_R_) across precipitation and potassium (P) gradients (the latter denoted by a color gradient).

**Figure 4:**
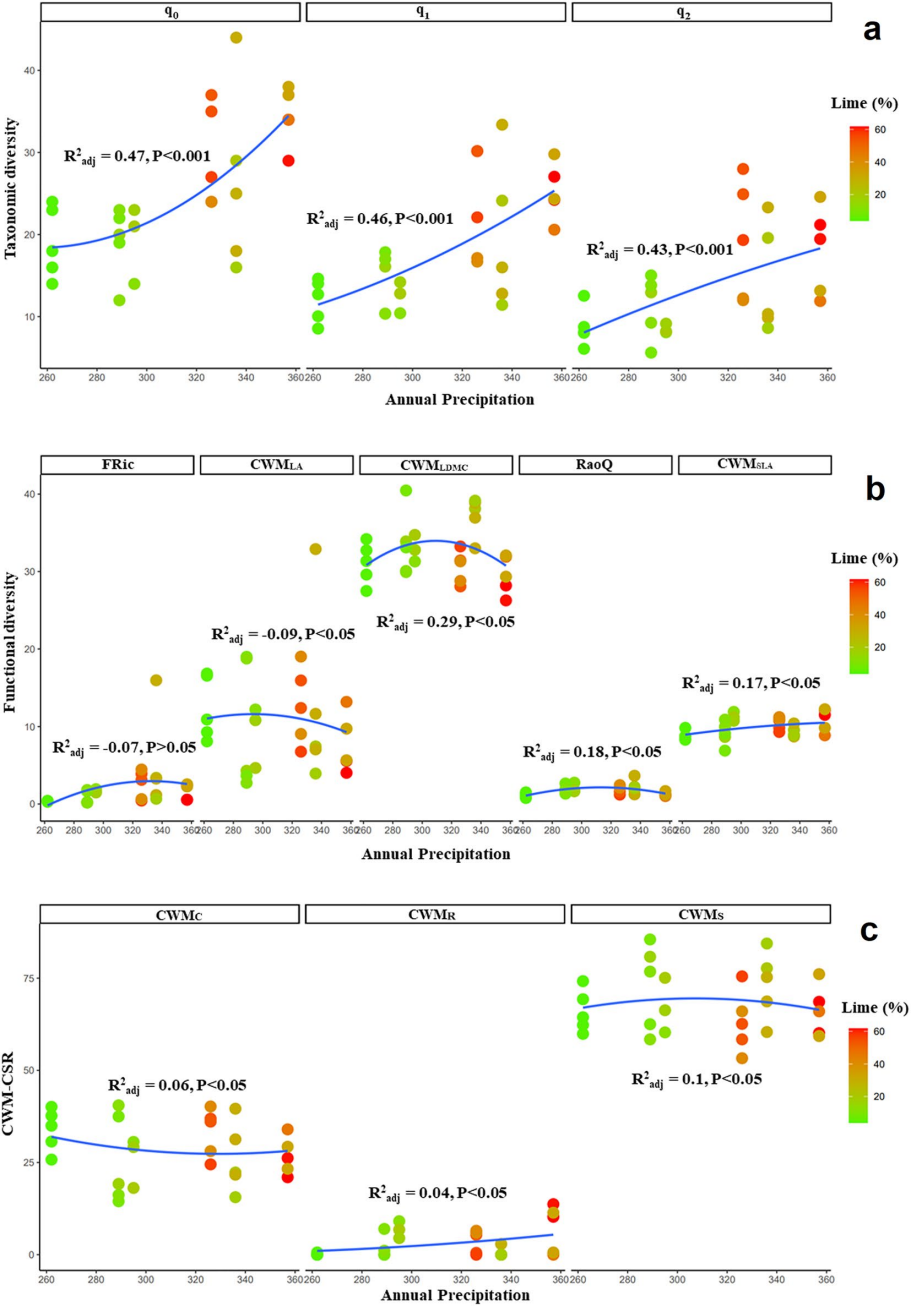
Alpha diversity of communities hosting *D. polylepis* subsp. *polylepis*: (**a**). taxonomic diversity (species richness, q_0_; Shannon’s diversity, q_1_, Simpson’s diversity, q_2_), (**b**). functional diversity (FRic, RaoQ, CWM_LDMC_, CWM_SLA_, CWM_LA_), and (**c**). plant ecological strategy diversity (C, S, R strategies; CWM_C_, CWM_S_, CWM_R_) across precipitation and lime gradients (the latter denoted by a color gradient).

**Figure 5:**
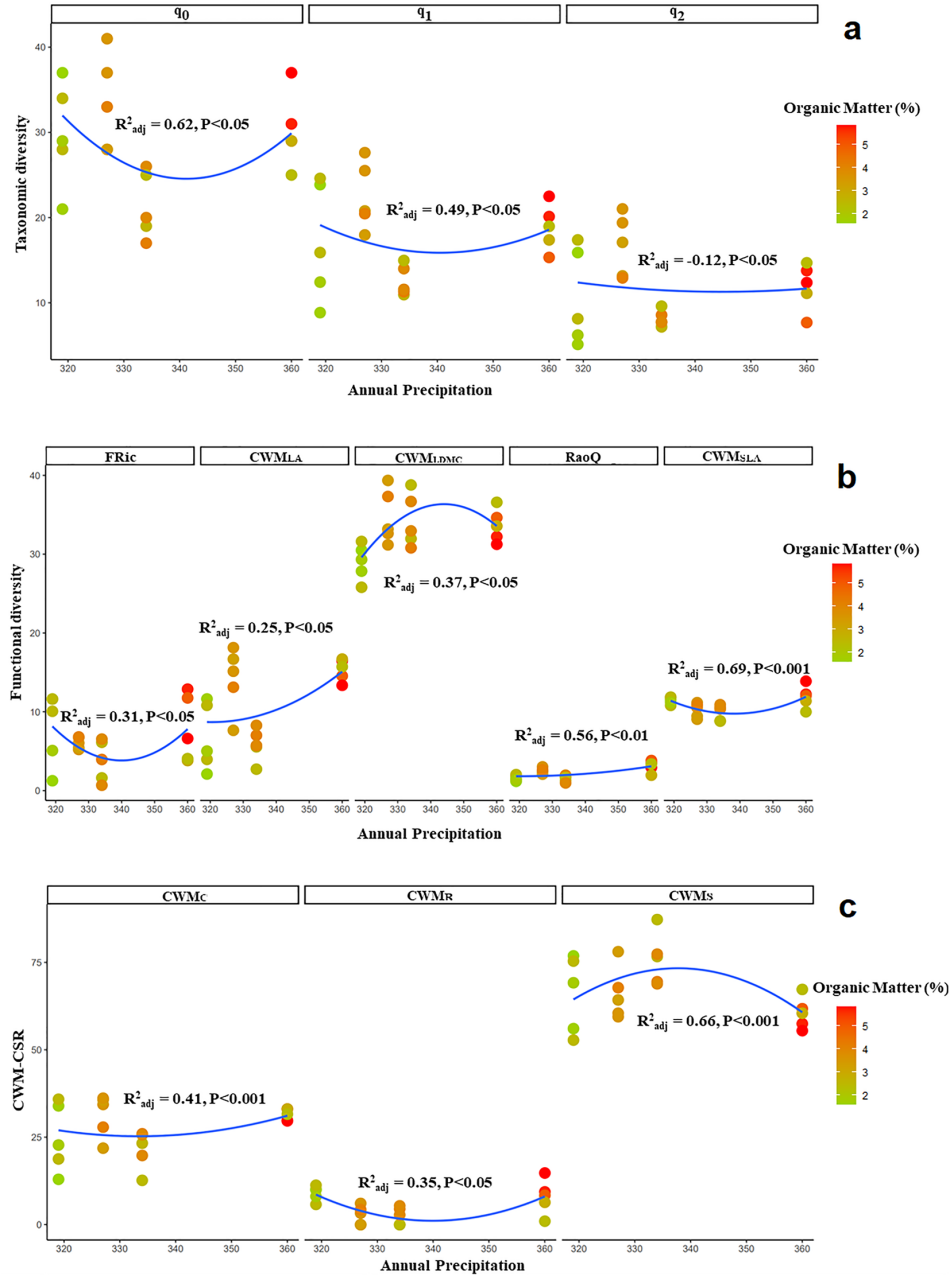
Alpha diversity of communities hosting *D. pseudocrinitus*: (**a**). taxonomic diversity (species richness, q_0_; Shannon’s diversity, q_1_, Simpson’s diversity, q_2_), (**b**). functional diversity (FRic, RaoQ, CWM_LDMC_, CWM_SLA_, CWM_LA_), and (**c**). plant ecological strategy diversity (C, S, R strategies; CWM_C_, CWM_S_, CWM_R_) across precipitation and organic matter (the latter denoted by a color gradient).

Significant changes were observed in taxonomic diversity across precipitation and soil factors for the communities of three *Dianthus* taxa. The absolute ranges of annual precipitation for the different *Dianthus* communities were similarly restricted and low (323–388 mm, 262–357 and 319–360 mm, respectively; Figs. 3, 4, 5, respectively). Ranges of soil factors included low to high concentrations of potassium (376.2–965.6 mg/kg), a moderate to very high absolute range of lime content (4–61.5% calcium carbonate), and a wide range of organic matter concentrations (1.57–5.85%). Taxonomic diversity was highly variable in the communities of *D. polylepis* subsp. *binaludensis* and little change was observed across the precipitation gradient in these communities (Fig. 3a, Table S3). In contrast, taxonomic diversity in the communities of *D. polylepis* subsp. *polylepis* exhibited directional changes along the gradient with more positive, relatively strict (and statistically well-supported) variations than the communities of *D. polylepis* subsp. *binaludensis* (Fig. 4a, Table S3). Hence, the variations of taxonomic diversity in the communities of *D. polylepis* subsp. *polylepis* are associated with the changes in precipitation across gradients. In contrast, *D. pseudocrinitus* communities exhibited a decrease in taxonomic diversity, especially under intermediate levels of precipitation and organic matter (see q_0_ and q_1_ panels in Fig. 5a and Table S3).

Functional indices indicated significant nonlinear variations across precipitation and potassium, lime, and organic matter gradients in the communities of all three *Dianthus* taxa (Figs. 3b, 4b, 5b). Despite high variability in functional diversity, different trends were observed across environmental factors in all communities. In this regard, functional diversity indices including CWM_LA_ (R^2^ = 0.18, P < 0.05), RaoQ (R^2^ = 0.27, P < 0.05) and CWM_SLA_ (R^2^ = − 0.33, P < 0.05) peaked significantly at intermediate levels of precipitation and potassium gradients in the communities of *D. polylepis* subsp. *binaludensis*, whereas other indices (FRic and CWM_LDMC_) decreased at intermediate values of these environmental factors (Fig. 3b). Functional diversity (FRic and CWM_LA_) was highly variable in the communities of *D. polylepis* subsp. *polylepis.* However, a peak in RaoQ (R^2^ = 0.18, P < 0.05) and CWM_SLA_ (R^2^ = 0.17, P < 0.05) and a significant decrease in CWM_LDMC_ (R^2^ = − 0.09, P < 0.05) were observed under moderate levels of precipitation and lime gradients in these communities (Fig. 4b). In the communities of *D. pseudocrinitus*, CWM_LA_ (R^2^ = 0.25, P < 0.05), CWM_LDMC_ (R^2^ = 0.37, P < 0.05) and RaoQ (R^2^ = 0.56, P < 0.05), indices significantly increased under intermediate levels of precipitation and organic matter, whereas FRic (R^2^ = 0.31, P < 0.05) and CWM_SLA_ (R^2^ = 0.69, P < 0.05) indices showed decreases under moderate levels of precipitation and organic matter (Fig. 5b). In total, our results showed extensive variability for functional diversity indices in the communities of the *Dianthus* taxa, except for the communities of *D. polylepis* subsp. *polylepis* which exhibited little change in FD along the gradient and were characterized by extensive stress tolerance (Fig. 4b). Furthermore, in the communities of *D. pseudocrinitus*, stronger variations in functional diversity indices were observed across environmental gradients with more positive and significant effects of precipitation and organic matter on RaoQ, CWM_LA_, and CWM_LDMC_ (Fig. 5b).

The community-weighted C, S, and R strategy means also showed significant non-linear variations across precipitation and soil factor gradients for all communities of the *Dianthus* taxa. Accordingly, there was a significant increase in CWM_C_ (R^2^ = 0.14, P < 0.05), and CWM_R_ (R^2^ = 0.21, P < 0.05) at intermediate levels of precipitation and potassium gradients in the communities of *D. polylepis* subsp. *binaludensis*, whereas CWM_S_ exhibited extensive variability in relation to these environmental factors (Fig. 3c). However, these communities were nonetheless characterized by a predominance of the stress tolerance strategy. In the communities of *D. polylepis* subsp. *polylepis*, although CWM_C_ and CWM_R_ exhibited extensive variability across precipitation and lime gradients, little directional change was observed. Moreover, these communities were also characterized by extensive stress tolerance (Fig. 4c). Finally, strong and significant decreases were evident for CWM_C_ (R^2^ = 0.41, P < 0.001), CWM_R_ (R^2^ = 0.35, P < 0.05) and a peak in CWM_S_ (R^2^ = 0.66, P < 0.001) at intermediate levels of precipitation and organic matter for the communities of *D. pseudocrinitus* (Fig. 5c). Indeed, variation in the community-weighted means of C, S, R strategies (especially CWM_S_) across environmental factors was strongest in communities hosting *D. pseudocrinitus* with respect to those hosting the *D. polylepis* subspecies, with more positive and significant effects of precipitation and organic matter (Fig. 5c).

### Relative contribution of precipitation and soil factors to taxonomic and functional diversity in the *Dianthus* communities

**Figure 6:**
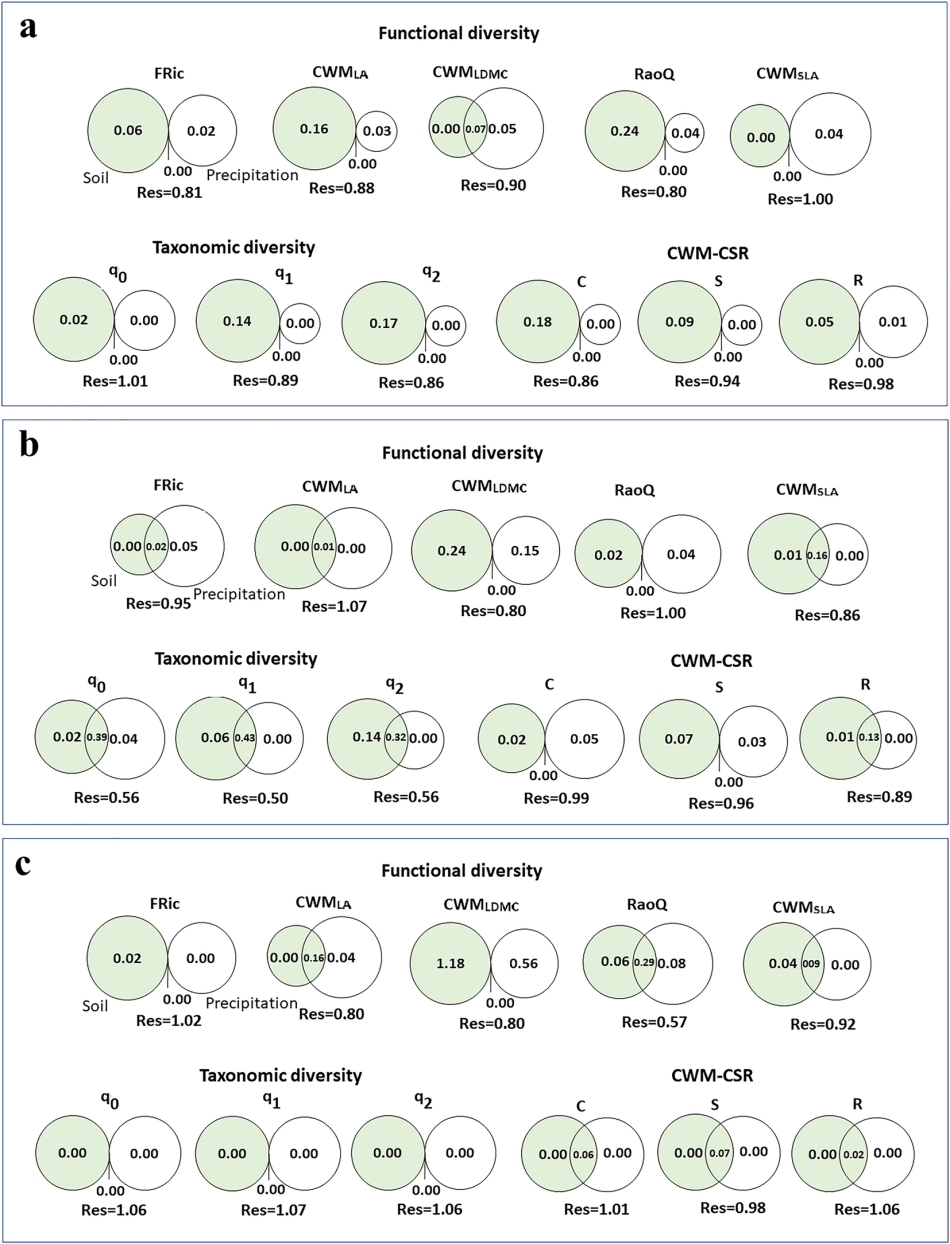
Variation partitioning (relative importance) of soil factors (green) and precipitation (in white) to taxonomic diversities (q_0_, q_1_, q_2_) and functional diversities (FRic, RaoQ, CWM_LDMC_, CWM_SLA_, CWM_LA_, CWM_C_, CWM_S_, CWM_R_) in the *Dianthus* communities. Note that for the communities of *D. polylepis* subsp. *binaludensis* (**a**) green represents soil potassium contents*,* for *Dianthus polylepis* subsp. *polylepis* (**b**) green represents lime contents, and for *Dianthus pseudocrinitus* (**c**) green represents organic matter contents. Values represent the adjusted R^2^-values.

Variation partitioning results (Fig. 6) demonstrate the relative importance of precipitation and soil factors (potassium, lime, and organic matter) to taxonomic and functional diversity in the communities of the *Dianthus* taxa. In the communities of *D. polylepis* subsp. *binaludensis*, a soil factor (potassium) explained higher contributions of variation in taxonomic (q_0_ = 2%, q_1_ = 14%, q_2_ = 17%) and functional diversity (CWM_LA_ = 16%, FRic = 6%, RaoQ = 24%) than precipitation, with the exceptions of CWM_LDMC_ and CWM_SLA_, and (CWM_C_ = 18%, CWM_S_ = 9%, CWM_R_ = 5%) (Fig. 6a). Based on these results, precipitation was not considered a strong factor in explaining the changes in biodiversity, especially taxonomic and functional diversity based on CSR traits, in the communities of *D. polylepis* subsp. *binaludensis*. Rather, precipitation had a stronger relationship with plant biodiversity in the communities of *D. polylepis* subsp. *polylepis* than in the communities of *D. polylepis* subsp. *binaludensis.* Accordingly, precipitation (Fig. 6b) explained a greater proportion of variation in taxonomic (q_0_ = 4%) and functional (FRic = 5%, RaoQ = 4%, CWM_C_ = 5%) diversity than these proportions in the communities of *D. polylepis* subsp. *binaludensis* (Fig. 6a,b). However, a soil factor (lime) also exhibited a large contribution of variation in taxonomic (q_1_ = 6%, q_2_ = 14%) and functional (CWM_LDMC_ = 24%, CWM_SLA_ = 1%; CWM_S_ = 7%, CWM_R_ = 1%) diversity in the communities of *D. polylepis* subsp. *polylepis*. Therefore, these results confirmed that precipitation and a soil factor (lime) are important factors in explaining the changes in biodiversity indices in communities hosting this subspecies. In the communities of *D. pseudocrinitus*, a soil factor (organic matter) and precipitation similarly explained a large proportion of variation in functional diversity (FRic = 2%, CWM_LDMC_ = 11.8%, CWM_SLA_ = 0.4%) and taxonomic diversity (CWM_LA_ = 4%, RaoQ = 8%). None of the soil and precipitation factors alone made significant contributions to variation in taxonomic diversity and functional diversity based on CWM_C_, CWM_S_, and CWM_R_ (all indices equaled zero; see Fig. 6c). However, soil and precipitation generally made a high contribution to functional diversity for CWM_C_ (6%), CWM_S_ (7%) and CWM_R_ (2%) indices (Fig. 6c). These results showed that despite soil and precipitation having constant and significant effects on functional diversity in the communities of *D. pseudocrinitus*, these factors were not influential in changing taxonomic diversity.

## Discussion

Our results provide evidence that biological diversity varies idiosyncratically in response to environmental gradients for communities hosting different species and subspecies of endemic *Dianthus*. Indeed, the hypothesis that differential variation in taxonomic and functional diversities occurs along climatic and soil factor gradients, with characteristic TD/FD responses for each *Dianthus* species community (Hypothesis 1) is supported. The results also support hypothesis 2, that adaptive specialization (low FD) is particularly evident in the harshest, arid habitats, converging on stress tolerance (evident for the communities of *D. polylepis* subsp. *polylepis*). Crucially, communities hosting different taxa exhibited contrasting diversity responses.

More specifically, both taxonomic diversity (q_0_, q_1_) and a range of functional traits and plant strategy scores varied significantly among the communities of the three *Dianthus* taxa. In this regard, the lowest values of taxonomic diversity indices were observed for the communities of *D. polylepis* subsp. *polylepis*. Although many studies confirm high species richness in mid-elevation ranges in different mountain regions, the communities of this taxon displayed a low taxonomic diversity in mountain steppes at elevational ranges of 1475 to 1894 m a.s.l.^68^. Thus, the low diversity of these communities could be related to the severe environmental conditions of this region. Indeed, the habitats of these taxa, especially in the Kashmar-Torbat ranges, are semi-arid and are affected by high temperature, low precipitation, high evaporation, and high salinity^69–73^, and the precipitation gradients in particular are evidently extreme (arid) and restricted in range (indeed, this was the case for all study taxa here). *D. polylepis* subsp. *binaludensis* communities exhibited higher taxonomic diversity, which may be related to specific environmental conditions in the Binalood mountains, such as a cold and arid climate, complex topography, and sequences of sedimentary, metamorphic, and igneous rock^33,73^. Previous studies also suggest increased taxonomic and functional diversity in different areas of the Binalood mountains^33,74,75^.

We observed almost the same functional diversity based on FRic, RaoQ, CWM_SLA_ indices for both subspecies that exhibited low functional diversity in comparison with the communities of *D. pseudocrinitus* (Fig. 2a,b). The harsh environmental conditions in the communities of both subspecies shape biodiversity patterns and limit functional diversity^76–78^. Indeed, Behroozian et al.^15^ demonstrated general functional convergence towards stress tolerance based on CSR strategies within the communities of these species, which clearly reflects the severe environments at the study sites.

Interestingly, we determined the highest levels of taxonomic diversity (q_0_ and q_1_) in the communities of *D. pseudocrinitus* currently impacted by high disturbance regimes. It has been suggested, in the form of the Humped-Back Model^79,80^ and the Intermediate Disturbance Hypothesis^81^ (IDH) that the greatest species diversity occurs at the mid-range of disturbance severity (see also^82,83^). Thus, an intermediate level of disturbance appears to operate in these communities. We also found that all three significant functional diversity indices (FRic, RaoQ, CWM_SLA_) were higher in the relatively disturbed communities of *D. pseudocrinitus* in comparison to the communities of the other taxa. This provides further support for the idea that the responses of both taxonomic and functional diversity to altered disturbance regimes underpin changes in biodiversity^80,84,85^.

Regarding plant community responses to precipitation and soil factor gradients, we found different responses of taxonomic and functional diversity to the precipitation and soil gradients, highlighting the importance of using these complementary diversity indices as ecological indicators to better understand plant community responses. Precipitation, potassium, lime, and organic matter were revealed as the main drivers of the overall biodiversity patterns. Our results indicated the different changes in taxonomic variability along soil and precipitation gradients in all communities (Figs. 3, 4, 5). However, different trends were observed across precipitation and soil factor gradients in taxonomic variability in the communities of *D. polylepis* subsp. *binaludensis* and *D. polylepis*. While the communities of *D. polylepis* subsp. *binaludensis* occurred over an extremely restricted precipitation range (differing between sites by only 65 mm per year), the communities hosting *D. polylepis* subsp. *polylepis* occurred over a slightly wider precipitation gradient (a 95 mm range but with lower, more arid absolute values) and all taxonomic diversity indices (q_0_, q_1_, q_2_) increased significantly and markedly with increasing lime; a response perhaps more evident due to the slightly broader precipitation gradient. This illustrates that ecosystems in which the rarest plants are found will not necessarily include a wide range of environmental variable values (and are thus not ideal systems for testing general hypotheses), especially for the rarest plants that may be specialist species restricted to extreme environments. However, these are precisely the ecosystems and species for which knowledge is most urgently required and for which observation of natural responses of established wild plants is valuable. Our results highlight that even extremely closely related taxa and their communities respond differently to aridity, suggesting that conservation responses should be considered on a case-by-case basis.

Stronger effects of precipitation than other climatic factors probably reflect the importance of water availability in the study area; precipitation is considered a critical limiting factor in semi-arid lands, considerably affecting plant diversity patterns^1^. On the other hand, potassium and lime significantly influenced biodiversity in the communities of *D. polylepis* subsp. *polylepis* and *D. polylepis* subsp. *binaludensis*, respectively. K and lime play a critical role in plant metabolism (particularly protein synthesis and photosynthesis), nutrient balance and uptake, and plant development, affecting biodiversity and ecosystem functions such as productivity and nutrient turnover rates^86–88^. Hence, it is suggested that the K and Ca cycles are nonetheless key determinants of plant diversity for these communities (see also^89–92^).

Diversity responses to environmental gradients, particularly precipitation, were nonlinear (Figs. 3b, 4b, 5b) and converged towards stress tolerance. This indicates the prevalence of low-yielding, functionally similar species^93,94^. Coexistence in these conditions is likely due to slight differences in functional traits and specific adaptations in response to each environmental factor^1,95,96^ followed by environmental filtering^97^. Both competition and facilitation, and thus the balance between these processes, are potentially involved^92^. The evident relationship between precipitation and lime with leaf dry matter content underpins the general stress-tolerance characteristics of these plant communities, probably related to an efficient nutrient conservation strategy of the species^98,99^. Indeed, leaves with a high dry matter content exhibit greater resistance to stressful conditions such as drought and freezing^100,101^ due to the accumulation of internal reserves that can be relied upon during extremes of environmental variability and the capacity to synthesize protective ‘chaperone’ proteins and sugars that protect metabolic machinery^1^. In contrast, the finding that greater precipitation and levels of edaphic factors favor less extreme adaptation and greater functional diversity agrees with studies relating greater FD to adaptations that allow survival following land use changes such as urbanization or agriculture^75^. This suggests that maintaining appropriate soil fertility and water availability regimes is likely to be key to the future persistence of the habitats of these rare, endemic *Dianthus* species.

## Conclusion

In conclusion, taxonomic and functional diversity responses to environmental factors are different between communities hosting two endemic and endangered *Dianthus* species (and sub-species) in mountain steppes. We found different responses of taxonomic and functional diversity to the precipitation and soil gradients, highlighting the importance of using these complementary diversity indices to assess communities of closely related endemic species in mountain ecosystems. Precipitation had a particularly strong effect, especially on functional diversity, suggesting that functional diversity could be particularly susceptible to climate changes, which should take precedence in future conservation planning. It would be valuable to conduct similar studies on plant biodiversity patterns of the communities of closely related mountain endemic species in other mountain regions, which respond distinctively to climatic and soil gradients. This would enhance our understanding of the taxonomic and functional patterns of these communities and help reduce the knowledge gap for mountain ecosystem biodiversity.

### Supplementary Information

Supplementary Tables.

## Data Availability

All data generated or analyzed during this study are included in this published article (Tables S1, S2 and S3 in supporting information).
