# Supplementary material for: Relative contributions of taxonomic and functional diversity to the assembly of plant communities hosting endemic Dianthus species in a mountain steppe
Source: Sci Rep. 2024 Mar 5;14:5471. doi: 10.1038/s41598-024-56099-x (PMC10915155; doi:10.1038/s41598-024-56099-x)
Supplement: Supplementary file 1 — Supplementary Tables. [file 41598_2024_56099_MOESM1_ESM.docx]

Supplementary Material

**Relative contributions of taxonomic and functional diversity to the assembly of plant communities hosting endemic *Dianthus* species in a mountain steppe**

Maryam Behroozian^1*^, Simon Pierce^2^, Hamid Ejtehadi^3^, Farshid Memariani^1,4^, Fahime Rafiee^3^, Mohammad Reza Joharchi^1^

^1^Herbarium FUMH, Ferdowsi University of Mashhad, Mashhad, Iran ([*maryam.behroozian94@gmail.com*](mailto:maryam.behroozian94@gmail.com); *joharchimr@yahoo.com*). ^2^Department of Agricultural and Environmental Sciences (DiSAA), University of Milan, Via G. Celoria 2, 20133 Milan, Italy ([*simon.pierce@unimi.it*](mailto:simon.pierce@unimi.it)). ^3^Quantitative Plant Ecology and Biodiversity Research Laboratory, Department of Biology, Faculty of Science, Ferdowsi University of Mashhad, Mashhad, Iran ([*hejtehadi@um.ac.ir*](mailto:hejtehadi@um.ac.ir); [f*ahime.rafiee@yahoo.com*](mailto:fahime.rafiee@yahoo.com)). ^4^Department of Range and Watershed Management, Faculty of Natural Resources and Environment, Ferdowsi University of Mashhad, Mashhad, Iran ([*memariani@um.ac.ir*](mailto:memariani@um.ac.ir)).

Corresponding author:

Maryam Behroozian; Herbarium FUMH, Ferdowsi University of Mashhad, Mashhad, Iran.

E–mail: *maryam.behroozian94@gmail.com*

**Table S1.** List of the 15 sites in the study area.

| Site code | Site name | Elevation range (m a.s.l.) | Longitude | Latitude |
| --- | --- | --- | --- | --- |
| S1 | Bezd | 1505-1525 | 60° 21′ 33.8″ | 35° 11′ 48.1″ |
| S2 | Kardeh dam | 1475-1491 | 59° 36′ 43.9″ | 36° 40′ 19.9″ |
| S3 | Kuhsorkh | 1493-1520 | 58° 29′ 25.1″ | 35° 24′ 19.1″ |
| S4 | Khowre- Kalat | 1776-1799 | 59° 52′ 45.1″ | 36° 38′ 20.0″ |
| S5 | Khomari pass | 1855-1893 | 59° 11′ 36.2″ | 35° 29′ 47.4″ |
| S6 | Balghour | 1794-1803 | 59° 34′ 29.6″ | 36° 48′ 18.7″ |
| S7 | Zoshk | 1793-1822 | 59° 12′ 38.2″ | 36° 19′ 54.1″ |
| S8 | Moghan | 1964-1974 | 59° 22′ 06.6″ | 36° 09′ 11.2″ |
| S9 | Dahane Jaji | 1796-1970 | 58° 57′ 22.0″ | 36° 28′ 45.8″ |
| S10 | Dizbad | 2007-2048 | 59° 18′ 00.7″ | 36° 05′ 01.0″ |
| S11 | Baharkish | 2179-2245 | 58° 40′ 03.7″ | 36° 41′ 31.2″ |
| S12 | Rein | 1871-1899 | 57° 02′ 27.6″ | 37° 24′ 06.8″ |
| S13 | Misino | 1647-1695 | 57° 29′ 44.9″ | 37° 54′ 53.3″ |
| S14 | Biu pass | 1664-1674 | 57° 11′ 56.4″ | 37° 21′ 31.7″ |
| S15 | Rakhtian | 1880-1923 | 57° 08′ 59.3″ | 37° 17′ 11.0″ |

**Table S2.** Checklist of vascular plants, their life-forms, chorology, threat category, and presence in habitats of three endemic *Dianthus* taxa in Khorassan- Kopet Dagh floristic province. Life-forms: Ch (chamaephytes), G.b (bulbous geophytes), G.c (cormous geophytes), G.t (tuberous geophytes), G.r (rhizomatous geophytes), G.p (parasitic geophytes), He (hemicryptophytes), Ph (phanerophytes), Th (therophytes). Chorotypes are according to Akhani (1998) and Memariani et al. (2016 a,b); IT: Irano-Turanian (for chorological subdivisions of IT elements, refer to Fig. 7); ES: Euro-Siberian; M: Mediterranean; SS: Sahara-Sindian; COS: Cosmopolitan; SCO: Sub-cosmopolitan; PL: Pluri-regional. Threat categories are given only for those species evaluated by Memariani et al. (2016b): CR (Critically Endangered), EN (Endangered), VU (Vulnerable), NT (Near Threatened), LC (Least Concern), and DD (Data Deficient).

| **NO** | **Families and species/infraspecific taxa** | **Life-form** | **Chorotype** | **Threat category** | **Habitats of *D*. *polylepis* subsp. *polylepis*** | **Habitats of *D.* *polylepis* subsp. *binaludensis*** | **Habitats of *D.* *pseudocrinitus*** |
| --- | --- | --- | --- | --- | --- | --- | --- |
|  | **Amaranthaceae** (including Chenopodiaceae) |  |  |  |  |  |  |
| 1 | *Noaea mucronata* (Forssk.) Asch. & Schweinf. | Ch | IT-ES-M |  | + | + | + |
|  | **Amaryllidaceae** |  |  |  |  |  |  |
| 2 | *Allium fibrosum* Regel | G.b | IT ^KK-E^ |  | + |  |  |
| 3 | *Allium kuhsorkhense* R.M.Fritsch & Joharchi | G.b | IT ^KK^ | NT | + | + |  |
| 4 | *Allium rubellum* M.Bieb. | G.b | IT ^Cauc.-Turk.^ |  | + |  | + |
| 5 | *Allium tenuicaule* Regel | G.b | IT ^KK-E^ |  | + | + |  |
| 6 | *Allium xiphopetalum* Aitch. & Baker | G.b | IT ^C & E^ |  | + | + |  |
|  | **Apiaceae** |  |  |  |  |  |  |
| 7 | *Astrodaucus orientalis* (L.) Drude | G.t | IT ^W & C^ |  |  | + |  |
| 8 | *Bunium afghanicum* Beauverd | G.t | IT ^KK-Afgh.^ |  | + | + |  |
| 9 | *Bunium cylindricum* (Boiss.& Hohen.) Drude | G.t | IT |  | + | + | + |
| 10 | *Bunium persicum* (Boiss.) B.Fedtsch. | G.t | IT ^C & E^ |  |  | + |  |
| 11 | *Bupleurum falcatum* L. subsp. *cernuum* (Ten.) Arcang. | Ch | IT |  | + | + | + |
| 12 | *Chaerophyllum khorassanicum* Czern. ex Schischk. | G.t | IT ^KK-Alborz^ |  |  | + |  |
| 13 | *Eryngium billardieri* F.Delaroche | He | IT ^C^ |  | + |  | + |
| 14 | *Eryngium bungei* Boiss. | He | IT ^C^ |  | + | + | + |
| 15 | *Ferula flabelliloba* Rech.f. & Aell. | He | IT ^KK^ | EN |  | + |  |
| 16 | *Ferula gumosa* Boiss. | He | IT ^C^ |  | + |  |  |
| 17 | *Ferula ovina* (Boiss.) Boiss. | He | IT ^C & E^ |  | + | + |  |
| 18 | *Galagania tenuisecta* (Regel & Schmalh.) M.G.Vassiljeva & Pim | G.t | IT ^KK-E^ |  |  |  | + |
| 19 | *Pimpinella aurea* DC. | He | IT ^W & C^ |  |  | + |  |
| 20 | *Pimpinella puberula* (DC.) Boiss. | Th | IT ^W & C^ |  | + |  |  |
| 21 | *Pimpinella tragium* Vill. | He | IT-ES-M |  | + | + | + |
| 22 | *Prangos latiloba* Korovin | He | IT ^C^ |  | + | + |  |
| 23 | *Pseudotrachydium vesiculosoalatum* (Rech.f.) Pimenov & Kljuykov | Ch | IT ^KK-Afgh.^ |  | + | + | + |
| 24 | *Scandix stellata* Banks & Sol. | Th | IT-M |  | + | + | + |
| 25 | *Torilis leptophylla* (L.) Reichenb. | Th | IT-ES |  |  |  | + |
| 26 | *Turgenia latifolia* (L.) Hoffm. | Th | IT-M |  | + | + | + |
| 27 | *Zeravschania aucheri* (Boiss.) M.Pimen. | He | IT ^C^ |  |  | + |  |
| 28 | *Zeravschania stricticaulis* (Rech.f.) Pimenov & Kljuykov | He | IT ^KK^ | VU | + |  |  |
|  | **Apocynaceae** |  |  |  |  |  |  |
| 29 | *Vincetoxicum pumilum* Decne. | He | IT ^KK^ | LC | + |  |  |
|  | **Asparagaceae** |  |  |  |  |  |  |
| 30 | *Muscari neglectum* Guss. ex Ten. | G.b | IT-ES-M |  | + |  |  |
|  | **Astraceae** |  |  |  |  |  |  |
| 31 | *Achillea biebersteinii* Afan. | He | IT |  |  |  | + |
| 32 | *Artemisia khorassanica* Podl. | Ch | IT ^KK^ | LC | + |  |  |
| 33 | *Artemisia kopetdaghensis* Krasch., Popov & Lincz. ex Poljakov | Ch | IT ^KK-Afgh.^ |  | + | + | + |
| 34 | *Carduus transcaspicus* subsp. *macrocephalus* (Arenes) Kazmi | Th | IT ^KK^ | NT |  |  | + |
| 35 | *Centaurea virgata* subsp. *squarrosa* (Willd.) Gugler | Ch | IT |  | + | + | + |
| 36 | *Chardinia orientalis* (L.) Kuntze | Th | IT ^W & C^ |  | + | + | + |
| 37 | *Cirsium bornmuelleri* Sint. ex Bornm. | He | IT ^KK^ | VU |  |  | + |
| 38 | *Cirsium sorocephalum* subsp. *congestum* (Fisch. & C.A.Mey. ex DC.) Yildiz, Dirmenci & Arabaci | He | IT |  |  | + |  |
| 39 | *Cirsium strigosum* (M. Bieb.) M.Bieb. | He | IT |  | + |  |  |
| 40 | *Cirsium vulgare* (Savi) Ten. | He | PL |  |  | + |  |
| 41 | *Cousinia chaetocephla* Kult. | He | IT ^KK^ | VU | + |  |  |
| 42 | *Cousinia concolor* Bunge | He | IT ^KK-Alborz^ |  |  | + |  |
| 43 | *Cousinia discolor* Bunge | He | IT ^KK^ | EN |  | + |  |
| 44 | *Cousinia elata* Boiss. & Buhse | He | IT ^KK-Alborz^ |  | + |  |  |
| 45 | *Cousinia eryngioides* Boiss. | He | IT ^C^ |  | + |  |  |
| 46 | *Cousinia freynii* Bornm. | He | IT ^KK^ | NT | + | + |  |
| 47 | *Cousinia hypopolia* Bornm. & Sing. | He | IT ^KK^ | VU |  |  | + |
| 48 | *Cousinia khorasanica* Djavadi & Attar | He | IT ^KK^ |  | + |  |  |
| 49 | *Cousinia microcarpa* Boiss. | He | IT ^KK-Afgh.^ |  |  |  | + |
| 50 | *Cousinia platyraphis* Kult. | He | IT ^KK^ | VU | + |  |  |
| 51 | *Cousinia smirnowii* Trautv. | He | IT ^KK-Alborz^ |  |  | + | + |
| 52 | *Cousinia stahliana* Bornm. & Gauba | He | IT ^KK^ | NT |  |  | + |
| 53 | *Crepis sancta* (L.) Bornm. | Th | IT-M |  |  |  | + |
| 54 | *Echinops heteromorphus* Bunge | He | IT ^KK^ | NT |  | + |  |
| 55 | *Echinops ritrodes* Bunge | He | IT ^C^ |  | + | + | + |
| 56 | *Echinops villosissimus* Bunge | He | IT ^C^ |  |  | + |  |
| 57 | *Gundelia tournefortii* L. | He | IT |  | + | + |  |
| 58 | *Helichrysum oocephalum* Boiss. | He | IT ^C^ |  | + | + | + |
| 59 | *Inula oculus-christi* L. | G.r | IT-ES-M |  |  |  | + |
| 60 | *Inula salicina* L. | G.r | IT-ES-M |  |  |  | + |
| 61 | *Jurinea sintenisii* Bornm. | Ch | IT ^KK^ |  | + | + |  |
| 62 | *Jurinea stenocalathia* Rech.f. | Ch | IT ^C^ |  | + | + |  |
| 63 | *Kalimeris altaicus* var. *canescens* (Nees) Sery. | He | IT-ES |  | + |  |  |
| 64 | *Klasea latifolia* (Boiss.) L.Martins | He | IT ^C^ |  | + | + | + |
| 65 | *Klasea leptoclada* (Bornm. & Sint.) L.Martins | Ch | IT ^KK^ | EN |  |  | + |
| 66 | *Koelpinia linearis* Pall. | Th | IT-SS |  | + |  | + |
| 67 | *Lactuca orientalis* (Boiss.) Boiss. | Ch | IT |  | + | + | + |
| 68 | *Lactuca persica* Boiss. | G.t | IT |  |  | + |  |
| 69 | *Lactuca serriola* L. | He | IT-ES-M |  |  |  | + |
| 70 | *Leontodon asperrimus* (Willd.) Boiss. ex Ball | He | IT ^W & C^ |  |  | + | + |
| 71 | *Leontodon kotschyi* Boiss. | He | IT ^Alborz^ |  |  |  | + |
| 72 | *Picnomon acarna* (L.) Cass. | He | IT-M |  |  |  | + |
| 73 | *Rhaponticum repens* (L.) Hidalgo | He | PL |  | + |  |  |
| 74 | *Sclerorhachis platyrachis* (Boiss.) Podlech ex Rech.f. | He | IT ^KK^ | LC | + |  | + |
| 75 | *Senecio paulsenii* O.Hoffm. subsp. *khorasanicus* (Rech.f. & Aellen) B.Nord. | G.r | IT ^C^ |  | + | + |  |
| 76 | *Tanacetum polycephalum* Sch.-Bip. | He | IT ^W & C^ |  |  |  | + |
| 77 | *Taraxacum sonchoides* (D.Don) Sehuitz Bip. | He | IT |  | + | + | + |
| 78 | *Taraxacum syriacum* Boiss. | He | IT |  |  | + |  |
| 79 | *Tragopogon coloratus* C.A.Mey. | He | IT ^W & C^ |  |  | + |  |
| 80 | *Tragopogon marginatus* Boiss. & Huet | He | IT ^C^ |  |  | + |  |
| 81 | *Varthemia persica* DC. | Ch | IT ^C^ |  | + | + |  |
| 82 | *Xeranthemum longopapposum* Fisch. & C.A.Mey | Th | IT |  | + |  |  |
|  | **Berberidaceae** |  |  |  |  |  |  |
| 83 | *Berberis integerrima* Bunge | Ph | IT |  | + | + |  |
| 84 | *Bongardia chrysogonum* (L.) Spach | G.t | IT |  |  | + | + |
|  | **Biebersteiniaceae** |  |  |  |  |  |  |
| 85 | *Biebersteinia multifida* DC. | G.t | IT |  | + | + |  |
|  | **Boraginaceae** |  |  |  |  |  |  |
| 86 | *Lappula barbata* (M.Bieb.) Gürke | Th | IT-M |  | + | + | + |
| 87 | *Lappula micricarpa* (Ledeb.) Gürke in Engler & Prantl. | Th | IT |  | + | + | + |
| 88 | *Lappula sinaica* (DC.) Ascherson ex Scheoinf. | Th | IT |  | + | + |  |
| 89 | *Myosotis stricta* Link | Th | IT-ES-M |  |  | + |  |
| 90 | *Onosma dichroantha* Boiss. | He | IT ^W & C^ |  |  | + | + |
| 91 | *Onosma longiloba* Bunge | He | IT ^KK-Alborz^ |  | + | + | + |
| 92 | *Paracaryum crista-galli* (Rech.f. & Riedl) D.Heller | He | IT ^KK-Afgh.^ |  | + |  |  |
| 93 | *Rochelia cardiosepala* Bunge | Th | IT |  | + | + | + |
| 94 | *Rochelia disperma* (L.f.) K.Koch. | Th | IT ^C^ |  | + |  |  |
| 95 | *Rochelia peduncularis* Boiss. | Th | IT |  | + |  |  |
| 96 | *Rochelia persica* Bunge ex Boiss. | Th | IT |  | + | + |  |
|  | **Brassicaceae** |  |  |  |  |  |  |
| 97 | *Alyssum baumgartnerianum* Bornm. | He | IT ^W & C^ |  |  | + | + |
| 98 | *Alyssum desertorum* Stapf | Th | IT-ES-M |  | + | + | + |
| 99 | *Alyssum inflatum* Nyár. | Th | IT ^C^ |  | + | + | + |
| 100 | *Alyssum lanceolatum* Baumgartner | Ch | IT ^KK-Afgh.^ |  |  | + |  |
| 101 | *Alyssum linifolium* Stephan ex Willd. | Th | IT |  | + |  |  |
| 102 | *Alyssum minus* Rothm. | Th | IT-M |  | + | + |  |
| 103 | *Alyssum mülleri* Boiss. & Buhse | He | IT ^C^ |  | + |  |  |
| 104 | *Alyssum polycladum* Rech.f. | He | IT ^C^ |  |  |  | + |
| 105 | *Alyssum* sp. | ? | ? |  |  | + |  |
| 106 | *Alyssum stapfii* Vierh. | Th | IT |  | + | + |  |
| 107 | *Alyssum szovitsianum* Fisch. & C.A.Mey. | Th | IT ^C^ |  | + | + | + |
| 108 | *Arabis nova* Vill. | Th | IT-ES-M |  | + | + | + |
| 109 | *Brassica elongata* Ehrh. | He | IT-ES-M |  | + |  |  |
| 110 | *Clypeola jonthlaspi* L. | Th | IT-M |  | + |  |  |
| 111 | *Conringia perfoliata* (C.A.Mey.) Busch | Th | IT ^W & C^ |  |  |  | + |
| 112 | *Conringia persica* Boiss. | Th | IT |  |  | + |  |
| 113 | *Crambe cordifolia* steven subsp. *kotschiana* (Boiss.) Jafri | He | IT |  | + | + |  |
| 114 | *Erysimum badghysi* (Korsh.) Lipsky ex N.Busch | He | IT ^KK-Afgh.^ |  |  | + |  |
| 115 | *Erysimum ischnostylum* Freyn & Sint. | He | IT ^KK-Afgh.^ |  |  |  | + |
| 116 | *Fibigia suffruticosa* (Vent.) Sweet | Ch | IT ^C^ |  |  | + |  |
| 117 | *Isatis brevipes* (Bunge) Jafri | Th | IT ^C & E^ |  |  | + |  |
| 118 | *Lepidium draba* L. subsp. *chalepense* (L.) P.Fourn. | He | IT-ES |  |  |  | + |
| 119 | *Lepidium litwinowii* (Lipsky) Al-Shehbaz | He | IT ^KK^ | NT |  |  | + |
| 120 | *Matthiola afghanica* Rech.f. | He | IT ^KK-Afgh.^ |  |  | + |  |
| 121 | *Matthiola alyssifolia* (DC.) Bornm. | He | IT ^C & E^ |  |  | + |  |
| 122 | *Noccaea perfoliata* (L.) Al-Shehbaz | Th | IT-ES-M |  | + |  | + |
| 123 | *Noccaea trinervia* Steud. | Ch | IT |  | + | + |  |
| 124 | *Parrya khorasanica* (Rech.f & Aellen) A.D. German & Al-Shehbaz | He | IT ^KK-E^ |  |  | + |  |
| 125 | *Strigosella africana* (L.) Botsch. | Th | IT-M-SS |  | + |  |  |
|  | **Caprifoliaceae** |  |  |  |  |  |  |
| 126 | *Cephalaria microcephala* Boiss. | He | IT ^C^ |  |  | + |  |
| 127 | *Lomelosia olivieri* (Coult.) Greuter & Burdet | Th | IT |  |  |  | + |
| 128 | *Lonicera bracteolaris* Boiss. & Buhse | Ph | IT ^C & E^ |  |  |  | + |
| 129 | *Lonicera iberica* M.Bieb. | Ph | IT ^Cauc.-Turk.^ |  |  |  | + |
| 130 | *Valerianella oxyrrhyncha* Fisch. & C.A.Mey. | Th | IT |  |  | + | + |
|  | **Caryophyllaceae** |  |  |  |  |  |  |
| 131 | *Acanthophyllum glandulosum* Buhse ex Boiss. | Ch | IT ^C & E^ |  | + | + | + |
| 132 | *Acanthophyllum pachystegium* Rech.f. | Ch | IT ^C^ |  |  |  | + |
| 133 | *Arenaria serpyllifolia* var. *macrocepala* Rech.f. | Th | PL |  |  |  | + |
| 134 | *Buffonia sintenisii* Freyn | Ch | IT ^KK-Afgh.^ |  | + |  |  |
| 135 | *Cerastium dichotomum* L. subsp. *inflatum* Cullen | Th | IT |  | + | + | + |
| 136 | *Dianthus orientalis* subsp. *stenocalyx* (Boiss.) Rech.f. | Ch | IT ^C^ |  |  |  | + |
| 137 | *Dianthus polylepis* Bien. ex Boiss. subsp. *binaludensis* (Rech.f.) Vaezi & Behrooz. | Ch | IT ^KK^ | VU |  | + |  |
| 138 | *Dianthus polylepis* Bien. ex Boiss. subsp. *polylepis* | Ch | IT ^KK^ | VU | + |  |  |
| 139 | *Dianthus pseudocrinitus* Behrooz. & Joharchi | Ch | IT ^KK^ | CR |  |  | + |
| 140 | *Gypsophila antoninae* Scgisck. | He | IT ^KK^ | EN | + |  |  |
| 141 | *Holosteum umbellatum* L. subsp. *glutinosum* (M.Bieb.) Nyman | Th | IT |  | + |  |  |
| 142 | *Lepyrodiclis stellarioides* Schrenk. | Th | IT ^C^ |  |  |  | + |
| 143 | *Mesostemma kotschyana* (Fenzl ex Boiss.) Vved. | He | IT |  | + | + |  |
| 144 | *Minuartia hamata* (Hausskn.) Mattf. | Th | IT-M |  |  |  | + |
| 145 | *Minuartia meyeri* (Boiss.) Bornm. | Th | IT |  | + | + | + |
| 146 | *Silene chaetodonta* Boiss. | Th | IT |  | + |  |  |
| 147 | *Silene crispans* Litv. | He | IT ^KK-Alborz^ |  | + |  |  |
| 148 | *Silene cyri* Schischk. | He | IT-ES |  |  |  | + |
| 149 | *Silene indeprensa* Schischk. | Ch | IT ^KK^ |  | + |  |  |
| 150 | *Silene* *swertiifolia* Boiss. | He | IT ^W & C^ |  | + | + | + |
| 151 | *Stellaria alsinoides* Boiss & Buhse | Th | IT ^C & E^ |  |  | + |  |
|  | **Colchiaceae** |  |  |  |  |  |  |
| 152 | *Colchicum kotschyi* Boiss. | G.c | IT ^W & C^ |  |  | + |  |
|  | **Convolvulaceae** |  |  |  |  |  |  |
| 153 | *Convolvulus arvensis* L. | He | SCO |  |  | + | + |
| 154 | *Convolvulus lineatus* L. | He | IT-ES-M |  | + | + |  |
| 155 | *Convolvulus pseudocantabrica* Schrenk | He | IT ^KK-E^ |  |  |  | + |
| 156 | *Cuscuta epithymum* Murr. | Th | PL |  |  |  | + |
| 157 | *Cuscuta monogyna* Vahl | Th | IT-ES-M |  |  |  | + |
|  | **Crassulaceae** |  |  |  |  |  |  |
| 158 | *Rosularia subspicata* (Freyn) Boriss. | He | IT ^KK-E^ |  | + |  |  |
|  | **Cypraceae** |  |  |  |  |  |  |
| 159 | *Carex stenophylla* Wahlenb. | He | PL |  |  | + |  |
|  | **Ephedraceae** |  |  |  |  |  |  |
| 160 | *Ephedra intermedia* Schrenk & C.A.Mey. | Ph | IT ^C^ |  | + | + |  |
| 161 | *Ephedra major* Host | Ph | IT-ES-M |  |  |  | + |
|  | **Euphorbiaceae** |  |  |  |  |  |  |
| 162 | *Euphorbia aucheri* Boiss. | Th | IT ^C^ |  |  |  | + |
| 163 | *Euphorbia boissieriana* (Woron.) Prokh. | He | IT |  |  |  | + |
| 164 | *Euphorbia buhsei* Boiss. | He | IT |  | + |  |  |
| 165 | *Euphorbia bungei* Boiss. | He | IT ^W & C^ |  | + | + | + |
| 166 | *Euphorbia kopetdaghi* (Prokh.) Prokh. | He | IT ^KK^ | NT |  |  | + |
| 167 | *Euphorbia microsciadia* Boiss. | He | IT ^C^ |  | + | + |  |
| 168 | *Euphorbia spinidens* Bornm. ex Prokh. | Ch | IT ^KK-E^ |  |  |  | + |
| 169 | *Euphorbia szovitsii* Fisch. & C.A.Mey. | Th | IT |  | + | + | + |
|  | **Fabaceae** |  |  |  |  |  |  |
| 170 | *Astragalus ackerbergensis* Freyn & Sint. | He | IT ^KK^ | LC | + |  |  |
| 171 | *Astragalus basineri* Trautv. | He | IT ^KK-Afgh.^ |  | + | + |  |
| 172 | *Astragalus brevidens* Freyn & Sint. | He | IT ^KK^ | LC |  | + |  |
| 173 | *Astragalus citrinus* Bunge | He | IT ^C^ |  | + | + |  |
| 174 | *Astragalus crenatus* Schult. | Th | IT-M-SS |  | + |  |  |
| 175 | *Astragalus culminatus* Maassoumi, Kaz.Osaloo & Joharchi | He | IT ^KK^ | DD | + |  |  |
| 176 | *Astragalus dipelta* Bunge | Th | IT ^C^ |  |  | + |  |
| 177 | *Astragalus floccosus* Boiss. | Ch | IT ^C^ |  |  | + |  |
| 178 | *Astragalus gompholobium* Bunge | He | IT ^C^ |  | + |  |  |
| 179 | *Astragalus jolderensis* B.Fedtsch. | He | IT ^KK-Alborz^ |  |  | + | + |
| 180 | *Astragalus kashmarensis* Maassoumi & Podlech | He | IT ^KK^ |  | + |  |  |
| 181 | *Astragalus lycioides* Boiss. | Ch | IT ^C^ |  |  | + |  |
| 182 | *Astragalus masenderanus* Bunge | He | IT ^C^ |  | + | + |  |
| 183 | *Astragalus mercklinii* Boiss. & Buhse | He | IT ^C^ |  | + | + |  |
| 184 | *Astragalus neoassadianus* Rangbar | He | IT ^KK^ | VU |  |  | + |
| 185 | *Astragalus pendulinus* Popov & B.Fedtsch | He | IT ^KK^ | LC | + | + | + |
| 186 | *Astragalus raddei* Basil. | Ch | IT ^KK^ | NT | + |  | + |
| 187 | *Astragalus retamocarpus* Boiss. | He | IT ^C & E^ |  |  | + |  |
| 188 | *Astragalus schahrudensis* Bunge | He | IT ^KK-Alborz^ |  |  | + |  |
| 189 | *Astragalus* sp. | ? | ? |  |  |  | + |
| 190 | *Astragalus stalinskyi* Sirj. | Th | IT ^C & E^ |  |  | + |  |
| 191 | *Astragalus suluklensis* Freyn & Sint. | He | IT ^KK^ | LC | + | + | + |
| 192 | *Astragalus sumbari* Popov. | He | IT ^KK^ | LC | + |  |  |
| 193 | *Astragalus sympileicarpus* Rech.f. | He | IT ^KK^ | VU |  | + |  |
| 194 | *Astragalus turkmenorum* (Boiss.) Boriss. | Ch | IT ^KK^ | EN | + |  |  |
| 195 | *Astragalus verus* Olivier | Ch | IT ^C^ |  | + | + | + |
| 196 | *Astragalus zoshkensis* F.Ghahrem | Ch | IT ^KK^ | EN |  | + | + |
| 197 | *Hedysarum longipedunculatum* Ranjbar & Karamian | He | IT ^KK^ |  |  |  | + |
| 198 | *Hedysarum wrightianum* Aitch. & Baker | He | IT ^C^ |  | + | + |  |
| 199 | *Lathyrus inconspicuus* L. | Th | IT-M |  | + |  |  |
| 200 | *Medicago lupulina* L. | Th | PL |  |  |  | + |
| 201 | *Medicago sativa* L. | He | PL |  |  |  | + |
| 202 | *Onobrychis chorassanica* Bunge | He | IT ^KK-E^ |  |  |  | + |
| 203 | *Onobrychis cornuta* (L.) Desv. | Ch | IT |  | + |  | + |
| 204 | *Onobrychis sintenisii* Bornm. | He | IT ^KK-Alborz^ |  |  |  | + |
| 205 | *Onobrychis verae* Sirj. | He | IT ^KK-Afgh.^ | DD | + | + | + |
| 206 | *Oxytropis binaudensis* Vassilcz. | He | IT ^KK^ | DD |  | + |  |
| 207 | *Oxytropis kuchanensis* Vassilcz. | He | IT ^KK^ |  | + | + |  |
| 208 | *Oxytropis pseudosuavis* Maassoumi | He | IT ^C^ |  |  | + |  |
| 209 | *Trigonella subenervis* Rech.f. | He | IT ^KK^ | VU | + | + | + |
| 210 | *Vicia ervilia* (L.) Willd. | Th | IT-M |  | + |  |  |
| 211 | *Vicia peregrina* L. | Th | IT-ES-M |  |  | + |  |
| 212 | *Vicia subvillosa* (Ledeb.) Boiss. | G.r | IT ^C & E^ |  | + | + |  |
|  | **Geraniaceae** |  |  |  |  |  |  |
| 213 | *Geranium kotschyi* Boiss. | G.t | IT ^C^ |  | + | + |  |
|  | **Hypericaceae** |  |  |  |  |  |  |
| 214 | *Hypericum elongatum* Ledeb. | He | IT-M |  |  |  | + |
| 215 | *Hypericum helianthemoides* (Spach) Boiss. | He | IT ^C^ |  | + |  |  |
| 216 | *Hypericum scabrum* L. | He | IT ^W & C^ |  | + | + | + |
|  | **Iridaceae** |  |  |  |  |  |  |
| 217 | *Iris fosteriana* Aitch. & Baker | G.t | IT ^KK-Afgh.^ |  | + | + |  |
| 218 | *Iris kopetdagensis* (Vved.) B.Mathew & Wendelbo | G.t | IT ^KK-Afgh.^ |  | + |  |  |
| 219 | *Iris songarica* Schrenk | G.r | IT ^C & E^ |  |  | + |  |
|  | **Ixioliridaceae** |  |  |  |  |  |  |
| 220 | *Ixiolirion tataricum* (Pall.) Herb. | G.b | IT |  | + | + |  |
|  | **Lamiaceae** |  |  |  |  |  |  |
| 221 | *Clinopodium graveolens* (M.Bieb.) Kuntze | Th | IT-ES-M |  |  | + |  |
| 222 | *Dracocephalum kotschyi* Boiss. | Ch | IT ^C^ |  |  |  | + |
| 223 | *Hymenocrater elegans* Bunge. | Ch | IT ^KK-Alborz^ |  |  | + | + |
| 224 | *Hymenocrater oxyodontus* Rech.f. | Ch | IT ^C^ |  |  | + |  |
| 225 | *Hymenocrater platysteginus* Rech.f. | Ch | IT ^KK^ |  | + |  |  |
| 226 | *Lagochilus khorassanica* Zereatkar F.Ghahrem & Joharchi | Ch | IT ^KK^ |  | + |  | + |
| 227 | *Lamium amplexicaule* L. | Th | SCO |  | + |  |  |
| 228 | *Nepeta bracteata* Benth. | Th | IT ^C & E^ |  | + | + |  |
| 229 | *Nepeta glomerulosa* Boiss. subsp. *glomerulosa* | He | IT ^C^ |  | + |  |  |
| 230 | *Phlomis cancellata* Bunge | He | IT ^KK-Afgh.^ |  | + | + | + |
| 231 | *Phlomis herba-venti* L. | He | IT-ES-M |  |  |  | + |
| 232 | *Phlomoides binaludensis* Salmaki & Joharchi | He | IT ^KK^ | EN |  | + |  |
| 233 | *Phlomoides labiosa* (Bunge) Adylov, Kamelin & Makhm. | He | IT ^KK-E^ |  | + | + |  |
| 234 | *Phlomoides labiosiformis* (Popov) Adylov, Kamelin & Makhm. | He | IT ^C^ |  | + |  |  |
| 235 | *Salvia abrotanoides* (Kar.) Sytsma | Ch | IT ^C & E^ |  |  | + | + |
| 236 | *Salvia atropatana* Bunge | He | IT ^C^ |  |  |  | + |
| 237 | *Salvia chloroleuca* Rech.f. | He | IT ^KK^ | VU | + | + | + |
| 238 | *Scutellaria litwinowii* Bornm. | Ch | IT ^C^ |  | + |  |  |
| 239 | *Scutellaria pinnatifida* subsp. *alpina* (Bornm.) Rech.f. | Ch | IT ^C^ |  |  |  | + |
| 240 | *Stachys lavandulifolia* Vahl | He | IT ^C^ |  | + | + |  |
| 241 | *Stachys turcomanica* Trautv. | Ch | IT ^KK-Alborz^ |  |  |  | + |
| 242 | *Teucrium chamaedrys* L. | Ch | IT-ES-M |  |  |  | + |
| 243 | *Teucrium polium* L. | Ch | IT-M |  | + |  | + |
| 244 | *Thymus transcaspicus* Klokov. | Ch | IT ^KK^ | LC | + |  | + |
| 245 | *Thymus trautvetteri* Klokov & Desj.-Shost. | Ch | IT ^Cauc.-Turk.^ |  |  |  | + |
| 246 | *Ziziphora clinopodioides* Lam. subsp. *filicaulis* (Rech.f.) | Ch | IT-ES |  | + | + | + |
| 247 | *Ziziphora persica* Bunge | Th | IT |  |  |  | + |
| 248 | *Ziziphora tenuior* L. | Th | IT |  | + | + | + |
|  | **Liliaceae** |  |  |  |  |  |  |
| 249 | *Fritillaria gibbosa* Boiss. | G.b | IT ^C^ |  | + | + |  |
| 250 | *Gagea gageoides* (Zucc) Vved. | G.b | IT ^W & C^ |  | + |  |  |
| 251 | *Gagea kunawurensis* (Royle) Greuter | G.b | IT ^C & E^ |  | + | + | + |
| 252 | *Gagea reticulata* (Pall.) Schult. & Schult.f. | G.b | IT |  | + | + |  |
| 253 | *Gagea setifolia* Baker | G.b | IT ^C & E^ |  | + |  | + |
| 254 | *Tulipa montana* var. *chrysantha* (Boiss) Wendelbo | G.b | IT ^C^ |  |  |  | + |
| 255 | *Tulipa undulatifolia* var. *micheliana* (Hoog) Wilford | G.b | IT ^KK-Alborz^ |  | + |  | + |
|  | **Orobanchaceae** |  |  |  |  |  |  |
| 256 | *Leptorhabdos parviflora* (Benth.) Benth. | Th | IT ^C & E^ |  | + |  | + |
| 257 | *Orobanche alba* Stephan | G.p | IT-ES |  |  |  | + |
| 258 | *Orobanche* sp. | G.p | ? |  | + |  | + |
|  | **Papaveraceae** |  |  |  |  |  |  |
| 259 | *Fumaria vaillantii* Loisel. in Desv. | Th | IT-ES-M |  |  | + |  |
| 260 | *Papaver decaisnei* Hochst. & Steud. ex Boiss. | Th | IT |  |  | + |  |
| 261 | *Papaver dubium* L. | Th | PL |  | + | + | + |
| 262 | *Roemeria hybrida* (L.) DC. | Th | IT-M-SS |  |  |  | + |
| 263 | *Roemeria refracta* DC. | Th | IT |  | + |  |  |
|  | **Plantaginaceae** |  |  |  |  |  |  |
| 264 | *Plantago lanceolata* L. | He | IT-ES-M |  |  |  | + |
| 265 | *Veronica biloba* Schreb. | Th | IT |  | + |  |  |
| 266 | *Veronica campylopoda* Boiss. | Th | IT |  | + | + | + |
| 267 | *Veronica capillipes* Nevski | Th | IT ^C & E^ |  | + | + |  |
| 268 | *Veronica czerniacowskiana* Monjuschko | He | IT ^KK^ | VU |  | + |  |
| 269 | *Veronica hederifolia* L. | Th | IT-ES-M |  |  | + |  |
| 270 | *Veronica khorassanica* Czerniak. | Th | IT |  | + |  | + |
| 271 | *Veronica polita* Fries | Th | PL |  |  | + |  |
|  | **Plumbaginaceae** |  |  |  |  |  |  |
| 272 | *Acantholimon avenaceum* Bunge | Ch | IT ^KK^ | LC | + | + |  |
| 273 | *Acantholimon bodeanum* Bunge | Ch | IT ^KK-Alborz^ |  |  |  | + |
| 274 | *Acantholimon erinaceum* (Jaub. & Spach) Lincz. | Ch | IT ^C & E^ |  | + | + | + |
| 275 | *Acantholimon raddeanum* Czernjak. | Ch | IT ^KK-E^ |  | + |  |  |
| 276 | *Acantholimon spinicalyx* Koeie & Rech.f. | Ch | IT ^KK^ | VU | + |  |  |
|  | **Poaceae** |  |  |  |  |  |  |
| 277 | *Aegilops triuncialis* L. | Th | IT-M |  | + |  | + |
| 278 | *Agropyron cristatum* (L.) Gaertn. | He | PL |  | + |  |  |
| 279 | *Arrhrnatherum kotschyi* Boiss | He | IT |  |  | + |  |
| 280 | *Boissiera squarrosa* (Banks & Sol.) Nevski | Th | IT-M |  | + | + | + |
| 281 | *Bromus danthoniae* Trin. | Th | PL |  | + | + | + |
| 282 | *Bromus japonicus* Thunb. | Th | PL |  |  |  | + |
| 283 | *Bromus kopetdaghensis* Drobov | He | IT ^KK-Alborz^ |  | + | + | + |
| 284 | *Bromus lanceolatus* Roth | Th | IT-M |  | + |  |  |
| 285 | *Bromus oxyodon* Schrenk | Th | IT ^C & E^ |  |  | + |  |
| 286 | *Bromus pseudodanthoniae* Drobow | Th | IT ^C & E^ |  | + | + |  |
| 287 | *Bromus* sp. | ? | ? |  | + | + |  |
| 288 | *Bromus tectorum* L. | Th | PL |  | + | + | + |
| 289 | *Dactylis glomerata* L. | He | PL |  | + |  | + |
| 290 | *Elymus hispidus* (Opiz) Melderis | He | IT-ES-M |  |  | + | + |
| 291 | *Elymus longearistatus* (Boiss.) Tzvelev | He | IT ^C^ |  |  | + |  |
| 292 | *Elymus repens* (L.) Gould subsp. *elongatiformis* (Drobow) Melderis | He | IT-ES |  | + |  | + |
| 293 | *Elymus repens* (L.) Gould subsp. *repens* | He | PL |  |  |  | + |
| 294 | *Eremopoa persica* (Trin.) Roshev. | Th | IT-M |  |  |  | + |
| 295 | *Eremopyrum bonaepartis* (Spreng.) Nevski | Th | IT |  | + |  |  |
| 296 | *Eremopyrum orientale* (L.) Jaub. | Th | IT-M |  |  |  | + |
| 297 | *Festuca* sp. | ? | ? |  | + |  |  |
| 298 | *Festuca valesiaca* Gaudin | He | IT-ES |  | + | + | + |
| 399 | *Henrardia persica* (Boiss.) C.E.Hubb. var. *persica* | Th | IT ^W & C^ |  | + |  |  |
| 300 | *Heteranthelium piliferum* (Banks & Soland) Hochst. | Th | IT |  |  | + |  |
| 301 | *Koeleria macrantha* (Ledeb.) Schult. | He | PL |  |  |  | + |
| 302 | *Melica ciliata* L. | He | IT-ES-M |  |  |  | + |
| 303 | *Melica persica* Kunth susbp. *persica* | He | IT |  | + | + | + |
| 304 | *Phleum paniculatum* Huds. | Th | IT-ES-M |  |  |  | + |
| 305 | *Piptatherum holciforme* (M.Bieb.) Roem. & Schult. | He | IT-M |  | + |  |  |
| 306 | *Piptatherum laterale* (Regel) Roshev. | He | IT ^C & E^ |  |  | + |  |
| 307 | *Poa bacteriana* Roshev. | He | IT ^KK-E^ |  |  |  | + |
| 308 | *Poa bulbosa* L. | He | IT-ES-M |  | + | + | + |
| 309 | *Poa* sp. | ? | ? |  | + | + | + |
| 310 | *Poa versicolor* Besser subsp. *araratica* (Trautv.) Tzvelev | He | IT |  |  | + |  |
| 311 | *Rhizocephalus orientalis* Boiss. | Th | IT ^W & C^ |  | + |  |  |
| 312 | *Stipa arabica* Trin. & Rupr. | He | IT |  | + | + | + |
| 313 | *Stipa caucasica* Schmalh. | He | IT |  |  |  | + |
| 314 | *Stipa hohenackeriana* Trin. & Rupr. | He | IT |  | + | + | + |
| 315 | *Stipa holosericea* Trin. | He | IT ^Cauc.-Turk.^ |  |  | + | + |
| 316 | *Stipa lessingiana* Trin. & Rupr. | He | IT-ES |  |  |  | + |
| 317 | *Stipa richteriana* Kar. & Kir. | He | IT ^KK-E^ |  |  | + |  |
| 318 | *Taeniatherum caput-medusae* (L.) Nevski | Th | IT-ES-M |  | + | + | + |
| 319 | *Trisetum flavescens* (L.) P.Beauv. | He | IT-ES-M |  |  |  | + |
|  | **Polygonaceae** |  |  |  |  |  |  |
| 320 | *Polygonum paronychioides* C.A.Mey. | He | IT |  | + | + | + |
| 321 | *Rumex tianschanicus* Losinsk. | G.r | IT ^C & E^ |  |  | + |  |
|  | **Primulaceae** |  |  |  |  |  |  |
| 322 | *Androsace maxima* L. | Th | IT-ES-M |  | + |  | + |
|  | **Ranunculaceae** |  |  |  |  |  |  |
| 323 | *Ceratocephala testiculata* (Crantz) Besser | Th | IT-ES-M |  | + | + |  |
| 324 | *Nigella integrifolia* Regel | Th | IT ^C & E^ |  | + |  | + |
| 325 | *Ranunculus olgae* Regel | He | IT ^C & E^ |  | + |  |  |
| 326 | *Thalictrum isopyroides* C.A.Mey. | G.r | IT |  | + | + |  |
| 327 | *Thalictrum sultanabadense* Stapf | He | IT ^C^ |  | + | + | + |
|  | **Resedaceae** |  |  |  |  |  |  |
| 328 | *Reseda lutea* L. | He | IT-ES-M |  | + | + | + |
|  | **Rhamnaceae** |  |  |  |  |  |  |
| 329 | *Rhamnus pallasii* Fisch. & C.A.Mey. | Ph | IT-ES |  |  |  | + |
|  | **Rosaceae** |  |  |  |  |  |  |
| 330 | *Cotoneaster kotschyi* Klotz | Ph | IT |  |  |  | + |
| 331 | *Cotoneaster nummularioides* Pojark. | Ph | IT ^C & E^ |  |  |  | + |
| 332 | *Cotoneaster nummularius* Fisch. & C.A.Mey. | Ph | IT |  |  | + | + |
| 333 | *Prunus microcarpa* C.A.Mey. | Ph | IT ^W & C^ |  |  |  | + |
| 334 | *Prunus pseudoprostrata* (Pojark.)Rech.f. | Ch | IT ^KK-Alborz^ |  | + | + | + |
| 335 | *Prunus turcomanica* (Lincz.) Kitam. | Ph | IT ^KK^ |  | + | + | + |
| 336 | *Rosa beggeriana* Schrenk | Ph | IT ^C & E^ |  |  |  | + |
| 337 | *Rosa hemisphaerica* J.Herrmann | Ph | IT ^Cauc.-Turk.^ |  |  |  | + |
| 338 | *Rosa persica* Michx. ex Juss. | Ch | IT ^C & E^ |  | + | + | + |
| 339 | *Sanguisorba minor* Scop. | He | IT-ES-M |  | + | + | + |
|  | **Rubiaceae** |  |  |  |  |  |  |
| 340 | *Asperula arvensis* L. | Th | IT-ES-M |  | + |  | + |
| 341 | *Asperula glomerata* (M.Bieb.) Griseb. subsp. *turcomanica* (Pobed.) Ehrend. & Schönb.-Tem. | He | IT ^C & E^ |  | + | + | + |
| 342 | *Asperula setosa* Jaub. & Spach | Th | IT |  |  | + | + |
| 343 | *Callipeltis cucullaris* (L.) Rothm. | Th | IT-M |  |  | + | + |
| 344 | *Crucianella exasperata* Fisch. & C.A.Mey. | Th | IT |  | + | + | + |
| 345 | *Crucianella gilanica* Trin. subsp. *transcaspica* (Ehrend.) Ehrend. & Schönb.-Tem. | He | IT ^C & E^ |  | + | + | + |
| 346 | *Crucianella sintenisii* Bornm. | He | IT ^KK^ | VU |  | + | + |
| 347 | *Galium murale* (L.) All. | Th | IT-M |  | + |  |  |
| 348 | *Galium setaceum* Lam. | Th | IT-M |  |  | + | + |
| 349 | *Galium songaricum* Schrenk ex Fisch. & C.A.Mey. | Th | IT ^KK-E^ |  | + |  |  |
| 350 | *Galium* sp. | ? | ? |  |  | + |  |
| 351 | *Galium spurium* L. | Th | IT-ES-M |  | + | + | + |
| 352 | *Galium tricornutum* Dandy | Th | IT-ES-SS |  | + |  |  |
| 353 | *Galium verticillatum* Danth. ex Lam. | Th | IT-M |  | + |  |  |
| 354 | *Galium verum* L. | He | PL |  |  |  | + |
| 355 | *Rubia florida* Boiss. | Ch | IT ^C^ |  |  |  | + |
|  | **Rutaceae** |  |  |  |  |  |  |
| 356 | *Haplophyllum acutifolium* (DC.) G.Don | He | IT ^C & E^ |  |  | + |  |
|  | **Santalaceae** |  |  |  |  |  |  |
| 357 | *Thesium arvense* Horv. | G.r | IT-ES-SS |  |  |  | + |
| 358 | *Thesium kotschyanum* Boiss. | G.r | IT ^W & C^ |  | + |  | + |
|  | **Sapindaceae** |  |  |  |  |  |  |
| 359 | *Acer monspessulanum* subsp *turcomanicum* (Pojark.) Rech.f. | Ph | IT ^KK^ |  |  |  | + |
|  | **Scrophulariaceae** |  |  |  |  |  |  |
| 360 | *Linaria khorasanensis* Hamdi & Assadi | He | IT ^KK^ |  |  | + |  |
| 361 | *Verbascum cheiranthifolium* Boiss. | He | IT ^W & C^ |  | + | + | + |
| 362 | *Verbascum songaricum* Schrenk | He | IT |  |  | + |  |
| 363 | *Verbascum speciosum* Schrad. | He | IT-M |  |  | + | + |
|  | **Thymelaeaceae** |  |  |  |  |  |  |
| 364 | *Diarthron antoninae* (Pobed.) Kit Tam | Ch | IT ^KK^ |  |  |  | + |
| 365 | *Diarthron vesiculosum* C.A.Mey. | Th | IT |  | + |  | + |
|  | **Urticaceae** |  |  |  |  |  |  |
| 366 | *Parietaria judaica* L. | Ch | IT-ES-M |  |  |  | + |
|  | **Violaceae** |  |  |  |  |  |  |
| 367 | *Viola occulta* Lehm. | Th | IT |  |  |  | + |
|  | **Xanthorrhoeaceae** |  |  |  |  |  |  |
| 368 | *Eremurus olgae* Regel | G.r | IT ^KK-E^ |  | + | + |  |
| 369 | *Eremurus spectabilis* M.Bieb. | G.r | IT |  | + | + |  |
| 370 | *Eremurus stenophyllus* (Boiss. & Buhse) Baker | G.r | IT ^C & E^ |  | + | + |  |

**Table S3.** Results of linear and nonlinear regression models on the effect of precipitation and soil factors on taxonomic (q_0_, q_1_, q_2_ Hill) and functional (Functional richness (FRic), Rao’s quadrate entropy (RaoQ), Community-Weighted mean Specific Leaf Area (CWM_SLA_), Community-Weighted mean Leaf Dry Matter Content (CWM_LDMC_), Community-Weighted mean Leaf Area (CWM_LA_), Community-Weighted mean of competitive ability (CWM_C_), Community-Weighted mean of stress-tolerance (CWM_S_) and Community-Weighted mean of ruderality (CWM_R_) indices) diversity along precipitation and soil factors gradient in the communities of *Dianthus* taxa.

| lm | species | response | explanatory | R^2^ | P | AIC |
| --- | --- | --- | --- | --- | --- | --- |
| linear | *D. polylepis* subsp. *binaludensis* | q_0_ | annual precipitation + K | -0.01457 | 0.4552 | 173.304 |
| nonlinear | *D. polylepis* subsp. *binaludensis* | q_0_ | annual precipitation + K | 0.05755 | 0.2677 | 172.9639 |
| linear | *D. polylepis* subsp. *binaludensis* | q_1_ | annual precipitation + K | 0.6399 | 1.82E-06 | 145.3399 |
| nonlinear | *D. polylepis* subsp. *binaludensis* | q_1_ | annual precipitation + K | 0.5129 | 0.000435 | 155.1428 |
| linear | *D. polylepis* subsp. *binaludensis* | q_2_ | annual precipitation + K | 0.07945 | 0.2198 | 149.5031 |
| nonlinear | *D. polylepis* subsp. *binaludensis* | q_2_ | annual precipitation + K | 0.1439 | 0.05934 | 145.8937 |
| linear | *D. polylepis* subsp. *binaludensis* | FRic | annual precipitation + K | -0.00985 | 0.4305 | 179.6906 |
| nonlinear | *D. polylepis* subsp. *binaludensis* | FRic | annual precipitation + K | 0.2479 | 0.03467 | 173.3847 |
| linear | *D. polylepis* subsp. *binaludensis* | CWM_LA_ | annual precipitation + K | 0.119 | 0.08368 | 517.3053 |
| nonlinear | *D. polylepis* subsp. *binaludensis* | CWM_LA_ | annual precipitation + K | 0.1846 | 0.07435 | 516.865 |
| linear | *D. polylepis* subsp. *binaludensis* | CWM_LDMC_ | annual precipitation + K | 0.1019 | 0.1054 | 162.9544 |
| nonlinear | *D. polylepis* subsp. *binaludensis* | CWM_LDMC_ | annual precipitation + K | 0.292 | 0.0193 | 158.1827 |
| linear | *D. polylepis* subsp. *binaludensis* | CWM_RoaQ_ | annual precipitation + K | 0.2021 | 0.02548 | 97.07954 |
| nonlinear | *D. polylepis* subsp. *binaludensis* | CWM_RoaQ_ | annual precipitation + K | 0.2761 | 0.02395 | 96.10157 |
| linear | *D. polylepis* subsp. *binaludensis* | CWM_SLA_ | annual precipitation + K | -0.00257 | 0.3947 | 106.4362 |
| nonlinear | *D. polylepis* subsp. *binaludensis* | CWM_SLA_ | annual precipitation + K | 0.3338 | 0.01059 | 97.05144 |
| linear | *D. polylepis* subsp. *binaludensis* | CWM_C_ | annual precipitation + K | 0.1405 | 0.06222 | 231.1574 |
| nonlinear | *D. polylepis* subsp. *binaludensis* | CWM_C_ | annual precipitation + K | 0.1447 | 0.02856 | 232.2725 |
| linear | *D. polylepis* subsp. *binaludensis* | CWM_S_ | annual precipitation + K | 0.05887 | 0.1848 | 229.6933 |
| nonlinear | *D. polylepis* subsp. *binaludensis* | CWM_S_ | annual precipitation + K | 0.2103 | 0.05517 | 226.6089 |
| linear | *D. polylepis* subsp. *binaludensis* | CWM_R_ | annual precipitation + K | 0.02257 | 0.291 | 188.6384 |
| nonlinear | *D. polylepis* subsp. *binaludensis* | CWM_R_ | annual precipitation + K | 0.01006 | 0.02716 | 186.8467 |
| linear | *D. polylepis* subsp. *polylepis* | q_0_ | annual precipitation +lime | 0.4443 | 0.000247 | 186.7658 |
| nonlinear | *D. polylepis* subsp. *polylepis* | q_0_ | annual precipitation +lime | 0.4777 | 0.000667 | 186.697 |
| linear | *D. polylepis* subsp. *polylepis* | q_1_ | annual precipitation +lime | 0.4047 | 0.000164 | 187.7885 |
| nonlinear | *D. polylepis* subsp. *polylepis* | q_1_ | annual precipitation +lime | 0.4668 | 0.000832 | 178.8299 |
| linear | *D. polylepis* subsp. *polylepis* | q_2_ | annual precipitation +lime | 0.4375 | 0.000288 | 171.3294 |
| nonlinear | *D. polylepis* subsp. *polylepis* | q_2_ | annual precipitation +lime | 0.3943 | 0.003256 | 175.0656 |
| linear | *D. polylepis* subsp. *polylepis* | FRic | annual precipitation +lime | 0.04776 | 0.2073 | 144.7159 |
| nonlinear | *D. polylepis* subsp. *polylepis* | FRic | annual precipitation +lime | 0.07632 | 0.2191 | 145.5287 |
| linear | *D. polylepis* subsp. *polylepis* | CWM_LA_ | annual precipitation +lime | -0.07036 | 0.894 | 450.1138 |
| nonlinear | *D. polylepis* subsp. *polylepis* | CWM_LA_ | annual precipitation +lime | -0.07036 | 0.7661 | 452.2218 |
| linear | *D. polylepis* subsp. *polylepis* | CWM_LDMC_ | annual precipitation +lime | 0.2035 | 0.02225 | 150.7339 |
| nonlinear | *D. polylepis* subsp. *polylepis* | CWM_LDMC_ | annual precipitation +lime | 0.3169 | 0.01147 | 148.0962 |
| linear | *D. polylepis* subsp. *polylepis* | CWM_RoaQ_ | annual precipitation +lime | -0.03378 | 0.5788 | -12.79 |
| nonlinear | *D. polylepis* subsp. *polylepis* | CWM_RoaQ_ | annual precipitation +lime | 0.1766 | 0.06675 | -9.82632 |
| linear | *D. polylepis* subsp. *polylepis* | CWM_SLA_ | annual precipitation +lime | 0.1419 | 0.0564 | 96.07357 |
| nonlinear | *D. polylepis* subsp. *polylepis* | CWM_SLA_ | annual precipitation +lime | 0.1684 | 0.01728 | 99.92337 |
| linear | *D. polylepis* subsp. *polylepis* | CWM_C_ | annual precipitation +lime | 0.1135 | 0.08473 | 161.1103 |
| nonlinear | *D. polylepis* subsp. *polylepis* | CWM_C_ | annual precipitation +lime | 0.0619 | 0.2511 | 164.3605 |
| linear | *D. polylepis* subsp. *polylepis* | CWM_S_ | annual precipitation +lime | 0.009941 | 0.3373 | 202.0093 |
| nonlinear | *D. polylepis* subsp. *polylepis* | CWM_S_ | annual precipitation +lime | 0.04029 | 0.3054 | 202.8029 |
| linear | *D. polylepis* subsp. *polylepis* | CWM_R_ | annual precipitation +lime | 0.03597 | 0.2417 | 203.9904 |
| nonlinear | *D. polylepis* subsp. *polylepis* | CWM_R_ | annual precipitation +lime | 0.1022 | 0.05394 | 205.8073 |
| linear | *D. pseudocrinitus* | q_0_ | annual precipitation +OM | 0.2019 | 0.1287 | 135.3539 |
| nonlinear | *D. pseudocrinitus* | q_0_ | annual precipitation + OM | 0.6259 | 0.0577 | 137.7353 |
| linear | *D. pseudocrinitus* | q_1_ | annual precipitation + OM | -0.06849 | 0.6823 | 130.2495 |
| nonlinear | *D. pseudocrinitus* | q_1_ | annual precipitation + OM | 0.4912 | 0.02266 | 130.8693 |
| linear | *D. pseudocrinitus* | q_2_ | annual precipitation + OM | 0.8184 | 0.0916 | 124.7456 |
| nonlinear | *D. pseudocrinitus* | q_2_ | annual precipitation + OM | -0.1185 | 0.7386 | 126.7297 |
| linear | *D. pseudocrinitus* | FRic | annual precipitation + OM | -0.02426 | 0.4763 | 112.9995 |
| nonlinear | *D. pseudocrinitus* | FRic | annual precipitation + OM | 0.3147 | 0.04432 | 106.4581 |
| linear | *D. pseudocrinitus* | CWM_LA_ | annual precipitation + OM | -0.01035 | 0.424 | 112.0108 |
| nonlinear | *D. pseudocrinitus* | CWM_LA_ | annual precipitation + OM | 0.2552 | 0.07619 | 107.4079 |
| linear | *D. pseudocrinitus* | CWM_LDMC_ | annual precipitation + OM | 0.1954 | 0.06122 | 307.8729 |
| nonlinear | *D. pseudocrinitus* | CWM_LDMC_ | annual precipitation + OM | 0.3726 | 0.02463 | 304.3933 |
| linear | *D. pseudocrinitus* | CWM_RoaQ_ | annual precipitation + OM | 0.4339 | 0.003081 | 42.04682 |
| nonlinear | *D. pseudocrinitus* | CWM_RoaQ_ | annual precipitation + OM | 0.5594 | 0.002141 | 38.53277 |
| linear | *D. pseudocrinitus* | CWM_SLA_ | annual precipitation + OM | 0.07884 | 0.1933 | 68.88743 |
| nonlinear | *D. pseudocrinitus* | CWM_SLA_ | annual precipitation + OM | 0.6869 | 0.000186 | 48.79946 |
| linear | *D. pseudocrinitus* | CWM_C_ | annual precipitation + OM | 0.1377 | 0.1819 | 138.9028 |
| nonlinear | *D. pseudocrinitus* | CWM_C_ | annual precipitation + OM | 0.4094 | 0.00618 | 141.5453 |
| linear | *D. pseudocrinitus* | CWM_S_ | annual precipitation + OM | 0.017 | 0.3358 | 150.3138 |
| nonlinear | *D. pseudocrinitus* | CWM_S_ | annual precipitation + OM | 0.3541 | 0.02992 | 143.4105 |
| linear | *D. pseudocrinitus* | CWM_R_ | annual precipitation + OM | -0.06375 | 0.657 | 120.9482 |
| nonlinear | *D. pseudocrinitus* | CWM_R_ | annual precipitation + OM | 0.6635 | 0.000313 | 99.42555 |
